# Pulsed Electric Field (PEF) Treatment Results in Growth Promotion, Main Flavonoids Extraction, and Phytochemical Profile Modulation of *Scutellaria baicalensis* Georgi Roots

**DOI:** 10.3390/ijms26010100

**Published:** 2024-12-26

**Authors:** Kajetan Grzelka, Adam Matkowski, Grzegorz Chodaczek, Joanna Jaśpińska, Anna Pawlikowska-Bartosz, Wojciech Słupski, Dorota Lechniak, Małgorzata Szumacher-Strabel, Segun Olorunlowu, Karolina Szulc, Adam Cieślak, Sylwester Ślusarczyk

**Affiliations:** 1 Department of Pharmaceutical Biology and Biotechnology, Division of Pharmaceutical Biology and Botany, Wrocław Medical University, 50-367 Wrocław, Poland; kajetan.grzelka@student.umw.edu.pl (K.G.); pharmaceutical.biology@wp.eu (A.M.); 2 Botanical Garden of Medicinal Plants, Wrocław Medical University, 50-367 Wrocław, Poland; joanna.jaspinska@umw.edu.pl (J.J.); anna.pawlikowska-bartosz@umw.edu.pl (A.P.-B.); 3 Bioimaging Laboratory at Łukasiewicz Research Network—PORT Polish Center for Technology Development, 54-066 Wrocław, Poland; grzegorz.chodaczek@port.lukasiewicz.gov.pl; 4 Department of Pharmacology, Wrocław Medical University, 50-367 Wrocław, Poland; wojciech.slupski@umw.edu.pl; 5 Department of Genetics and Animal Breeding, Poznań University of Life Sciences, Wołyńska 33, 60-637 Poznań, Poland; dorota.lechniak@up.poznan.pl; 6 Department of Animal Nutrition, Poznań University of Life Sciences, Poznań, Wołyńska 33, 60-637 Poznań, Poland; malgorzata.szumacher@up.poznan.pl (M.S.-S.); olorunlowusegunabraham@gmail.com (S.O.); 7 Department of Animal Breeding and Product Quality Assessment, Poznań University of Life Sciences, Zlotniki, ul. Słoneczna 1, 62-002 Suchy Las, Poland; karolina.szulc@up.poznan.pl

**Keywords:** electroporation, eutectic solvents, flavonoids, hydroponics

## Abstract

This study aims to explore the effect of pulsed electric field (PEF) treatment as a method very likely to result in reversible electroporation of *Scutellaria baicalensis* Georgi underground organs, resulting in increased mass transfer and secondary metabolites leakage. PEF treatment with previously established empirically tailored parameters [E = 0.3 kV/cm (U = 3 kV, d = 10 cm), t = 50 µs, N = 33 f = 1 Hz] was applied 1–3 times to *S. baicalensis* roots submerged in four different Natural Deep Eutectic Solvents (NADES) media (1—choline chloride/xylose (1:2) + 30% water, 2—choline chloride/glucose (1:2) + 30% water, 3—choline chloride/ethylene glycol (1:2), and 4—tap water (EC = 0.7 mS/cm). Confocal microscopy was utilized to visualize the impact of PEF treatment on the root cells in situ. As a result of plant cell membrane permeabilization, an extract containing major active metabolites was successfully acquired in most media, achieving the best results using medium 1 and repeating the PEF treatment twice (baicalein <LOQ, baicalin 12.85 µg/mL, wogonin 2.15 µg/mL, and wogonoside 3.01 µg/mL). Wogonin concentration in NADES media was on par with the control (plants harvested on the day of the experiment, ultrasound-mediated methanolic extraction, C_wogonin_ = 2.15 µg/mL). After successful extraction, PEF treatment allowed the plants to continue growing, with the lowest survival rate across treated groups being 60%. Additionally, an enhancement in plant growth parameters (length and fresh mass of the roots) and significant changes in the *S. baicalensis* root phytochemical profile were also observed.

## 1. Introduction

*Scutellaria baicalensis* roots (*Scutellariae baicalensis radix*) is a traditional Chinese herbal drug, listed among the most important for use in numerous directions, such as jaundice, hepatitis, autoimmune disorders, viral infections, central nervous system disorders, and many more [1]. The herb has also been included in other pharmacopeias, such as the Chinese [2], Japanese [3], and European [4] ones, as well as having an ISO norm (ISO 4564:2023) [5]. The major active compounds of this herb are flavones—baicalein and wogonin and their respective glucuronides, baicalin and wogonoside. This field-cultivated plant requires a few seasons to have the roots mature before harvest. Therefore, any approach that would facilitate obtaining these bioactives is useful and important to meet the high demand. Electroporation of biological membranes by the pulsed electric field (PEF) treatment is currently successfully used in many fields of medicine and pharmacy. For example, electroporation and chemotherapy are combined to promote drug delivery directly into cancer cells using electrical pulses [6]. Electroporation is also used to introduce nucleic acids or proteins into living cells with the aim of transient or stable transfection or the modification of cell function [7]. Plant protoplast gene electrotransfer has been successfully used to transform several crop species, such as corn, rice, wheat, soybean, and rye [8]. Finally, PEF treatment is also used to facilitate the extraction of active compounds from plant materials. An example of utilizing irreversible electroporation is the extraction of steroid alkaloids from potato peels [9]. One of the studies proposed using pulsed electric field (PEF) treatment as a method to evaluate resistance to oxidative stress induced by hydrogen peroxide on carrots. The specific treatment increased total carotenoid content and enhanced antioxidant activity. This study also highlighted improvements in cell viability and reductions in nitric oxide (NO) production under oxidative stress conditions [10]. Other researchers used PEF treatment on tomatoes, which resulted in increased concentrations of carotenoids such as lutein, lycopene, and β-carotene. This enhancement was attributed to the stress response induced by ATP release, which promoted metabolic activity [11]. In another study, PEF treatment was used as a pretreatment for subcritical water extraction of the quercetin (flavonoid) in onion [12]. This research established optimal electric field intensities and pulse durations for maximizing flavonoid content without significant degradation of bioactive compounds. Another study indicated that PEF treatment enhanced the release of anthocyanins and related antioxidants in grape juice [13]. PEF treatment increased the bioavailability and antioxidant capacity of these compounds, suggesting its application in nutraceutical production. PEF-induced oxidative stress in potatoes led to the accumulation of a phenolic compound, chlorogenic acid. This response was part of the plant’s defense mechanism, highlighting PEF treatment’s role in stimulating phenolic biosynthesis pathways. This study focuses on flavonoid pathways: while previous studies broadly assessed secondary metabolites, such as phenolics and carotenoids, the current study emphasizes the precise role of PEF treatment in activating flavonoid biosynthesis pathways, particularly the transcription factors (e.g., MYB, bHLH) and enzymatic reactions driving flavonoid synthesis. In another study, electroporation increased the efficiency of extraction of carotenoids from tomato peel by 39% in a mixture of hexane/ethanol/acetone (50:25:25) [11], and the content of environmentally harmful hexane in the extraction mixture was reduced without affecting the extraction efficiency. A study conducted on white mulberry *Morus alba* L. showed increased extraction of polyphenols using PEF treatment compared to the conventional maceration method [14]. Additionally, the use of PEF treatment significantly increased the antioxidant activity of basil, salvia, and tea leaf extracts [15,16,17]. It was also found that extraction using PEF treatment is the most effective method of obtaining protein from *Chlorella vulgaris* Beijerinck, compared to ultrasonic extraction or the freeze–thaw method. Protein extracted from microalgae for food, personal care products, and cosmetics must be of high purity and require the use of extraction techniques free of aggressive solvents, e.g., n-hexane or acetonitrile. Additionally, a few days after electroporation, *Chlorella* cells began to grow at the same rate as non-electroporated cells, which was not observed for other methods [18]. Successful PEF-mediated extraction of root active metabolites was also documented in the case of *Panax ginseng* C.A.Mey., resulting in higher compound yield and increased antioxidant activity [19]. The above examples prove that this study explores the universal impact of PEF treatment across a broader range of species, aiming to generalize its potential in enhancing flavonoid production. Also, our previous research done on aeroponic cultures of *Scutellaria baicalensis* Georgi seedlings showed an increased content of root flavonoids after PEF treatment, paired with a positive effect on root growth [20].

Natural Deep Eutectic Solvents (NADESs) are a fast-developing alternative to classical solvent extraction based on harmful solvents such as alcohols that damage living cells [21]. In this study, a comprehensive PEF-enhanced extraction procedure is documented. Moreover, several combinations of NADESs have been studied for extraction to avoid using more aggressive solvents and keep the plant roots alive. Building on our previous research on the application of PEF treatment to the roots of *S. baicalensis* seedlings, it was established that optimal treatment parameters for promoting plant growth—resulting in significantly higher average fresh and dry masses compared to the control—were E = 1.25 kV/cm, t = 25 ms, and N = 40. However, the living plants subjected to more severe treatment conditions (e.g., E = 5 kV/cm, t = 100 μs, N = 10, likely resulting in irreversible electroporation) are likely to perish [20].

Flavonoids, as a main group of *S. baicalensis* compounds, are classified as secondary metabolites and biostimulants. They play a key role in plant growth, but also in seed germination, flowering, and defense by inducing resistance against certain biotic and abiotic stresses. Flavonoids are important as signal compounds to communicate with rhizosphere microbes. The application of pulsed electric field (PEF) technology significantly impacts the biosynthesis of flavonoids in plants by inducing biochemical responses, mainly due to oxidative stress and secondly membrane disruption mechanisms [22]. PEF treatment generates reactive oxygen species (ROS) in plant cells, which act as signaling molecules. These signals activate transcription factors like MYB and bHLH, crucial in regulating the genes involved in phenolic and flavonoid biosynthesis [23]. This leads to the synthesis of enzymes, such as phenylalanine ammonialyase, which is one of the key enzymes acting between primary and secondary metabolism and catalyzes the deamination of L-phenylalanine to *trans*-cinnamic acid, and ammonia phenoloxidases and peroxidases, which catalyze the formation of flavonoids and other phenolic compounds [24,25]. The second proposed mechanism of action is that as a result of PEF treatment and cell membrane disruption via electroporation, ATP is released. It then acts as a secondary signal, further amplifying the stress response. This triggers metabolic pathways, such as the phenylpropanoid pathway, which is directly involved in flavonoid production [26]. The oxidative stress and cellular injury caused by PEF treatment may also elevate levels of secondary messengers like ethylene and jasmonic acid [23]. These compounds are known to enhance secondary metabolite production, including flavonoids, as a part of the plant’s defense mechanisms. PEF treatment also improves the recovery of flavonoids by increasing cell permeability. This facilitates the extraction of these compounds when PEF treatment is combined with other techniques, such as ultrasound or solvent-based methods, enhancing yield and bioactivity [27]. In this context, pulsed electric field (PEF)-based green processes have been investigated as a pretreatment to enhance industrial oil extraction processes. This innovative technology presents the potential to improve mass transfer under milder time and temperature conditions, reducing energy and solvent consumption.

However, over the course of three years of experiments, the treatment conditions have been carefully optimized, including adjustments to PEF parameters and the properties of the electroporation medium. This refined methodology has not only ensured the survival of the plants but also led to an improvement in their growth under specific treatment conditions. Unlike studies primarily focusing on increasing the extraction yield, this study incorporates the optimization of PEF parameters to minimize cellular damage while maximizing flavonoid biosynthesis and facilitating extraction from living plants, or so-called “root milking”. This approach balances efficiency with plant cell integrity, which is critical for sustainable applications. These distinctions make the current research a foundational contribution to understanding and harnessing PEF treatment’s potential for tailored plant metabolic enhancement. Most importantly, an extract containing major active metabolites in the electroporation medium was obtained, which, paired with an observed increase in conductivity of the electroporation medium, is clear evidence of permeabilization of the root cell membranes taking place [28,29].

## 2. Results and Discussion

### 2.1. Confocal Microscopy

**Figure 1:**
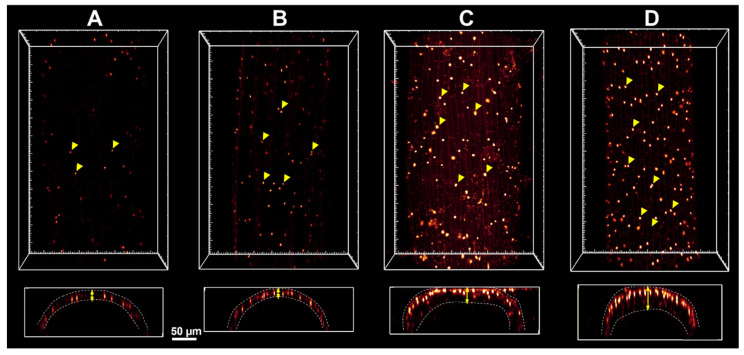
Three-dimensional confocal microscopy images of PI-stained *S. baicalensis* root sample after PEF administration of the following E: (**A**)—control, (**B**)—1 kV/cm, (**C**)—3 kV/cm, (**D**)—7.5 kV/cm in H_2_O. The yellow arrowheads indicate the PI-stained nuclei and the arrows in the lower row images indicate the thickness of penetrated layer depending on the applied voltage (Table S1).

**Figure 2:**
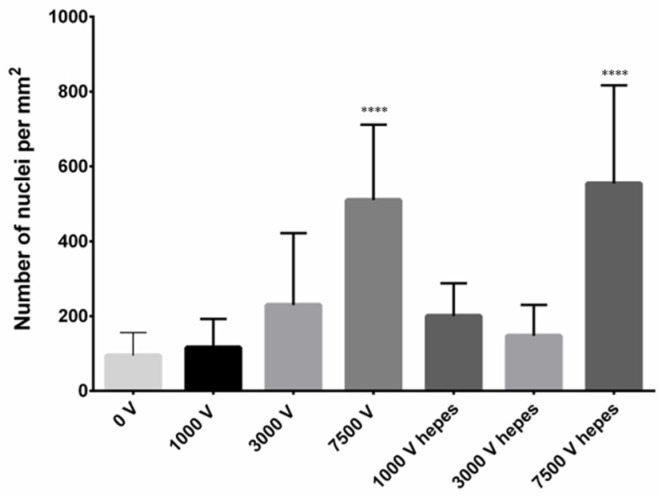
The number of nuclei per mm^2^ of the root section with means and standard deviations (±SD). Successfully stained by PI depending on electric field strength (V) and usage of buffer (HEPES). Statistically significant responses (**** *p* < 0.00005; ANOVA with Tukey’s multiple comparison test). Differences and tendencies when compared to the control group. More details are included in the Supplementary Materials (Tables S1 and S2).

Electroporation has been extensively studied, with the majority of research focusing on its application in animal cells [30]. The potential of applying methods used on mammalian tissue, namely PI staining, to evaluate PEF treatment’s impact on plants was explored in this study [31,32]. In a preliminary experiment conducted on three-week-old *S. baicalensis* seedlings using small-scale equipment, the effects of electroporation on *S. baicalensis* root tissue were successfully recorded and visualized. It was found that fluorescence and the number of stained root cell nuclei predictably increased with applied electric field strength (Figure 1 and Figure 2), confirming this method’s applicability in a PEF impact evaluation in plants. Observations of increased solvent conductivity after the PEF application further support root cell permeabilization. Notably, the results were largely unaffected by the choice of electroporation medium (either KCl solution or HEPES buffer) used.

### 2.2. Compound Extraction from Living S. baicalensis Roots

**Figure 3:**
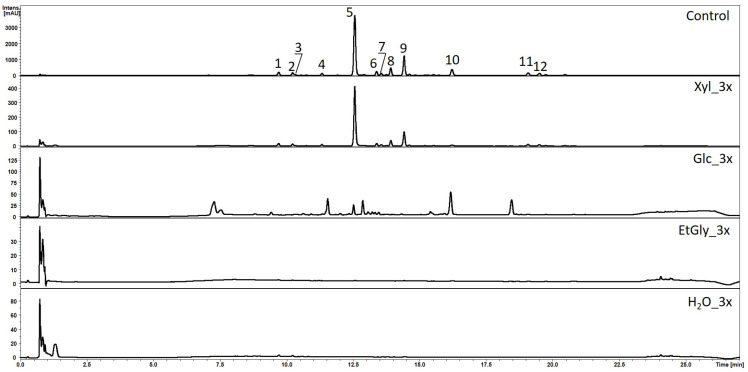
HPLC chromatograms (λ = 280 nm) of *S. baicalensis* methanolic (control) and NADES extracts; PEF treatment repeated 3 times; subgroups named according to Table 1; main peaks numbered according to Table 2 numeration.

Obtaining active compounds from plants in an environmentally friendly way is a vital aim of modern science. Classic extraction procedures mostly rely on toxic, volatile, and highly flammable organic solvents, e.g., methanol. Therefore, greener, more sustainable, and eco-friendly solvents, such as eutectic mixtures, are increasingly being considered [33,34]. However, using such compounds takes significantly more time due to their generally lower extraction efficiency. To enhance this process, we combined these solvents with PEF treatment, which is commonly used to enhance mass transfer and facilitate extraction from plant material [15,17,19,27]. Researchers also point to the potential of this method, specifically in the extraction of polyphenols [11,12]. In this study, three NADES media and tap water were used as conductive–extractive media during PEF treatments (Table 1). HPLC-MS qualitative analysis revealed that the extraction of *S. baicalensis* root flavonoids using PEF into NADES media was successful (Figure 3, Table 2). However, since the concentrations of compounds were generally low, in order to compare them with the control extracts, we decided to take into account solely the content of the main flavonoids, of which wogonoside and wogonin turned out to be the most abundant.

**Figure 4:**
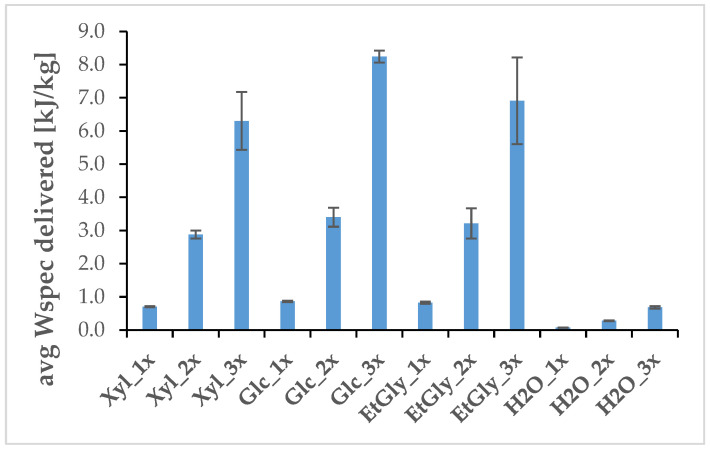
The average (Avg) specific energy input (Wspec) delivered to *S. baicalensis* roots during PEF treatment. The error bars indicate the standard deviation (±SD).

The negligible amounts of individual compounds in the extract can be attributed to the young age of the plants used in the experiment (six weeks old), as pharmacopeial raw material—*Scutellariae baicalensis rhizoma*—is typically recommended to be harvested and extracted when plants are at least several years old. Due to the trace amounts of baicalin and baicalein in the NADES extracts, they were excluded from the comparison. The main differentiating factor between treatments was the total specific energy input (Wspec) applied to the roots in each treatment (Figure 4), calculated as reported in other works in this field [35,36].

**Figure 5:**
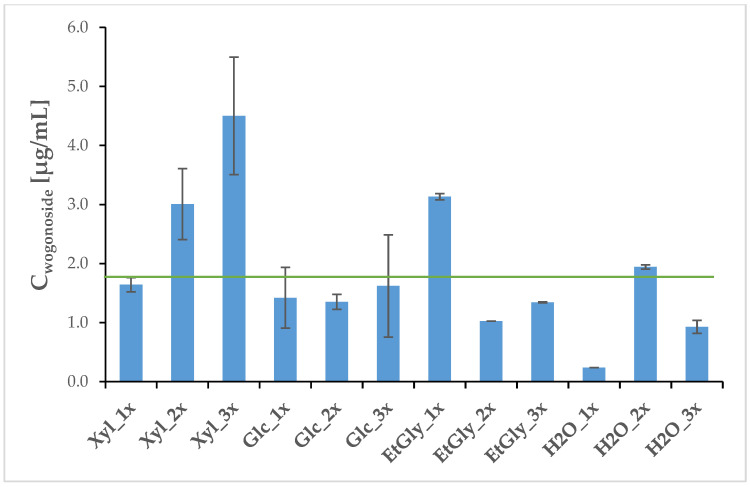
Average concentrations of wogonoside in electroporation media after PEF treatment. The green line indicates the average concentration across treated groups (1.85 μg/mL). The error bars indicate the standard deviation (±SD).

**Figure 6:**
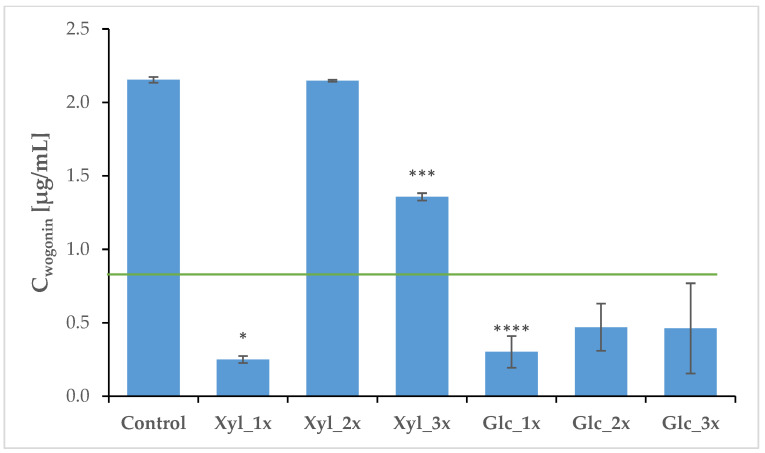
Average concentrations of wogonin in electroporation media after PEF treatment. The green line indicates the average concentration across treated groups (0.83 μg/mL). Symbols correspond to statistically significant (**** *p* < 0.00005; *** *p* < 0.0005 * *p* < 0.05 *t*-test results for independent groups) differences and tendencies when compared to the control group. The error bars indicate the standard deviation (±SD).

In the case of wogonoside, the control extract yielded a concentration of 41.02 µg/mL, significantly surpassing the treated groups (the average concentration across them was 2.09 µg/mL). As a result, the control extract is not included in the chart. Wogonoside extraction efficiency was evidently lower in treated subgroups when compared to the traditional methanolic extraction method (extraction control). This is likely because the presented treatment was intentionally mild and minimally invasive, preserving plant viability by periodically permeabilizing roots. In contrast, the conventional method employed in the control extraction involves root mincing and ultrasound-mediated methanolic extraction, which irreversibly breaks cell membranes to maximize compound release. The milder approach in this study preserved cell membrane integrity and physiological functions, supporting the goal of maintaining plant survival, enabling the performance of observations under less invasive conditions, and potentially allowing for re-extraction of the plants within a short timeframe, which is the main advantage of the presented method. The statistically significant differences between the control and treated groups were confirmed by the Mann–Whitney U test (details are included in Supplementary Table S3) since the distribution of the variables was not normal (*p* = 0.0048). Among the NADES extracts, the Xyl_3x treatment was the most effective, yielding 4.5 µg/mL of this compound, while the H_2_O_1x was the least efficient in its extraction, resulting in a concentration of 0.24 µg/mL (Figure 5, Table 3). This trend is reflected by Wspec delivered to the roots. Statistical analysis predictably revealed that there are statistically important differences between the control and all treated groups (*t*-test for independent groups *p* < 0.00000 across the board). Additionally, there was a moderate positive correlation (*p* = 0.45 in Pearson’s and Kendall’s correlation tests; Z = 2.0 in the latter; details included in Supplementary Tables S4 and S5) between Wspec values and wogonoside concentrations in the NADES extracts. This suggests, as expected, that the extraction process was more successful when more energy was delivered per kg of treated mass via a pulsed electric field. Xyl_2x treatment was particularly effective in wogonin extraction, as it yielded 2.15 µg/mL of the compound, being on par with the control. On the other hand, when either the choline chloride (ChCl) and ethylene glycol (EtGly) mixture or tap water were used, wogonin’s extraction was unsuccessful. This is most likely due to the high polarity of these two solvents, which were successful in extracting a more polar glucuronide form of this compound (wogonoside). The average wogonin concentration across all samples was 0.55 µg/mL (Figure 6, Table 3). No significant correlation was initially observed between the values of Wspec and wogonin concentrations. However, after normalizing the data, a relationship similar to that seen with wogonoside emerged.

### 2.3. Impact of PEF Treatment on Plant Growth and Development

Over a three-week observation period after PEF treatment, more than half of all treated specimens remained healthy. None of the plants from the control group (sham treatment) died, indicating that NADES solvents on their own are not harmful to the plants and also reaffirming the successful adaptation of *S. baicalensis* to aeroponic cultivation. As for the treated plants, the survival rate was noted at 60% in the following groups: Xyl_2x, Xyl_3x, Glc_3x, EtGly_1x, EtGly_2x, EtGly_3x, H_2_O_1x, and H_2_O_2x. In all other cases, it was 80% (Table 4). When it comes to plant growth, in this study, the findings from our previous work were reinforced [20], which is in line with the hypothesis that applying PEF treatment at a certain intensity results in plant growth stimulation, but should the treatment be too severe, necrosis occurs. This phenomenon was widely reported by researchers who subjected seven-day-old *Arabidopsis thaliana* (L.) Heynh. seedlings to reversible electroporation. This resulted in a larger (almost twice as much under specific conditions) leaf surface area compared to the control. The process stimulated the growth of *A. thaliana*, with an electric field strength of 5 kV/cm for each of the applied treatments. At higher values, especially with simultaneous longer pulse duration (t) or a bigger number of pulses (N), plant growth gives way to necrosis associated with irreversible cell membrane permeabilization. The authors suggest that the growth stimulation is due to the induction of a stress response in the plant by electroporation, probably through the release of calcium ions from within the cells [37]. Such an effect of electroporation has already been confirmed in mammalian cells, specifically laboratory mice, in studies on the potential application of PEF treatment in melanoma therapy [31]. Other researchers have shown a positive effect on stimulating plant growth, potentially increasing product yield without the need for excessive use of growth stimulators [38].

**Figure 7:**
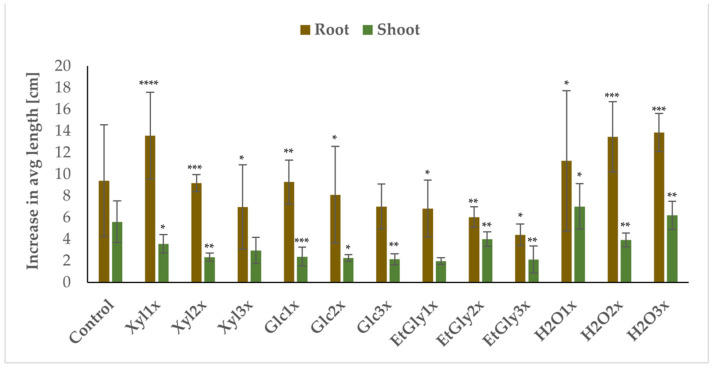
Average increase in *S. baicalensis* root and shoot length after PEF treatment over a three-week period. Symbols correspond to statistically significant (**** *p* < 0.00005; *** *p* < 0.0005 ** *p* < 0.005 * *p* < 0.05) *t*-test results for independent group differences and tendencies when compared to the control group. The error bars indicate the standard deviation (±SD).

In this case, the highest increase in *S. baicalensis* root growth was noted when tap water was used as an electroporation medium. Especially noteworthy is the fact that in the H_2_O_3x and Xyl_1x groups, the increase in average root size was nearly identical. This is likely linked to the Wspec that plants were subjected to—about 0.7 kJ/kg in both cases—and that exceeding such treatment intensity resulted in either unsuccessful growth stimulation in Xyl_2x and Glc_1x groups or diminished *S. baicalensis* root growth in all other cases. Aside from Wspec, this can also be attributed to osmotic stress in plants caused by xylose solutions. A high concentration of xylose in the medium creates a hypertonic environment, leading to water loss from plant cells. This simulates drought-like or high-salinity conditions, as the plant experiences water deficits and ion imbalances [39,40]. In cases of average shoot length increases, tap water (lowest intensity) treatments emerge as the most successful in growth promotion, with H_2_O_1x and H_2_O_3x being the only cases where the shoot length increase surpassed plants from the control group. That fact, paired with a very significant root length increase and a plant survival rate (PSR) of 80%, indicates that the H_2_O_3x treatment (Wspec = 0.68 kJ/kg) was optimal in promoting *S. baicalensis* growth and development. In cases where EtGly was used as an electroporation medium, both the plant survival rate (PSR) and plant growth and development (PGD) were among the lowest observed. This is evident in the EtGly_3x subgroup, which demonstrated the lowest increase in average root and shoot length among all treated groups, along with one of the lowest root dry masses for subsequent HPLC-MS analysis. These findings suggest that in this case, plant growth was impaired by necrosis induced by severe PEF treatment [15,17]. While the PSR was 60%, PGD reached only 20%, one of the lowest values recorded. This indicates that the secondary metabolism of plants in this subgroup may have been compromised, as the plants prioritized maintaining basic functions for survival. This observation, paired with the aforementioned low extraction yield, suggests that this solvent was not suitable for our purposes and should be either modified or disregarded in further studies (Figure 7, Table 4).

However, NADESs containing xylose are also found to be particularly effective for extracting phenolic and flavonoid compounds from natural sources [41], which is supported by aforementioned extraction results. This paired with a good plant response to PEF treatment in this medium makes xylose-based NADESs good candidates for further optimization. Overall, these results are in line with multiple reports suggesting that to achieve plant growth stimulation, specific and empirically adjusted treatment conditions must be provided; otherwise, electrostimulation either does not occur (too low intensity) or is masked by necrosis (too high intensity) [15,17].

### 2.4. Phytochemical Profile Modulation as a Result of PEF Treatment

PEF treatment, in addition to bolstering the extraction process, has an effect on the plant tissue and may serve as an abiotic stressor. Reversible electroporation has already been used as a stimulating factor in the production of specific substances by plants, such as active cytostatics in cell cultures of Chinese yew (*Taxus chinensis* Rehder & E.H. Wilson) [42]. Additionally, this process likely results in the production of reactive oxygen species that induce a stress response in the plant, which in turn may increase the production of antioxidants (e.g., polyphenols) [36,37,38,39]. The production of diverse phytochemicals, including flavonoids, to bolster antioxidant capacity is a common reaction to a comparable stressor: high salinity. This phenomenon is well documented as a way to induce oxidative stress in roots [23,41].

In our experiments, a PEF application resulted in significant shifts in the *S. baicalensis* root phytochemical profile. Flavonoid accumulation trends in plants treated with PEF differ in our experiment between aglycones (e.g., baicalein, wogonin) and glycosides (e.g., baicalin, wogonoside), probably due to their chemical structure and biosynthetic pathways. Higher relative increases in levels of particular aglycones, such as baicalein and wogonin, are likely due to PEF treatment’s ability to activate enzymes like β-glucosidase [26]. These enzymes hydrolyze glycosidic bonds in glycosides (e.g., baicalin, wogonoside), releasing the corresponding aglycone forms. Enhanced bioavailability of aglycones is likely due to them being more hydrophobic, facilitating their participation in cellular stress responses and ROS scavenging, the levels of which are typically elevated as a result of PEF treatment [22]. Moderate glycosides accumulate to a lesser extent, as PEF-induced stress typically prioritizes the mobilization of simpler bioactive molecules (aglycones). However, glycoside synthesis may be moderately promoted in cases where UDP-glucosyltransferase activity is upregulated, converting aglycones back into glycosides for storage. Higher electric field strengths tend to favor aglycone formation due to increased cellular disruption and enhanced enzymatic activity, whereas lower strengths may maintain a balance between glycoside stability and aglycone liberation. This can also be attributed to different parts of the plant exhibiting varying capacities for glycoside synthesis. For instance, wogonoside and baicalin levels may be naturally higher in root tissues, where glycosylation aids in long-term storage and stability. PEF-induced stress diverts metabolic flux towards phenylpropanoid and flavonoid biosynthetic pathways, favoring bioactive forms like aglycones that directly contribute to stress mitigation [43]. Another factor to be considered is the conductive–extractive medium itself. Natural Deep Eutectic Solvents (NADESs) are characterized by their ability to form stable mixtures with varying properties, including conductivity, influenced by the composition of the solvent and solutes.

**Figure 8:**
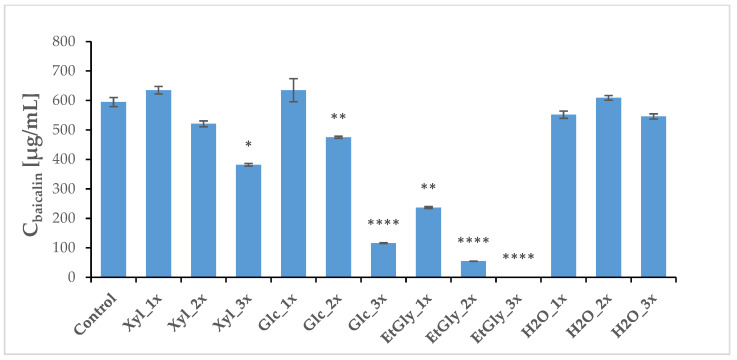
Average concentrations of main flavonoids in the *S. baicalensis* root extract—glucuronic acids, baicalin. Symbols correspond to statistically significant (**** *p* < 0.00005; ** *p* < 0.005 * *p* < 0.05) *t*-test results for independent groups. The error bars indicate the standard deviation (±SD).

**Figure 9:**
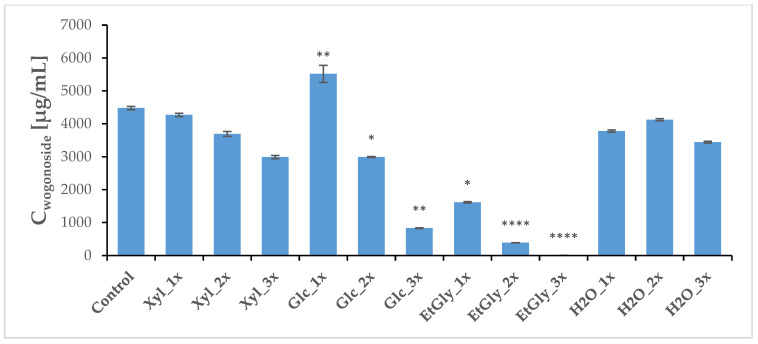
Average concentrations of main flavonoids in the *S. baicalensis* root extract—glucuronic acids, wogonoside. Symbols correspond to statistically significant (**** *p* < 0.00005; ** *p* < 0.005 * *p* < 0.05) *t*-test results for independent groups. The error bars indicate the standard deviation (± SD).

Three weeks after PEF treatment, the plants (both treated and sham-treated controls) were harvested, and a regular methanolic extract was prepared. HPLC-MS analysis revealed that the baicalin and wogonoside contents did not change very significantly. However, Glc_1x and Xyl_1x treatments resulted in a slight increase in their levels in comparison with the control (Figure 8 and Figure 9, Table 5). The aglycone content was most heavily influenced, which is in line with our previous study [20]. The ChCl: Xyl (1:2) + 30% water mixture (medium 1) emerged as the most consistent in promoting their content, after PEF application—an increase in both baicalein and wogonin concentrations was noted in most cases. However, it was the ChCl: EtGly (1:2) mixture (medium 3) that resulted in the most drastic change—a 3.7-fold increase—in wogonin content.

Solvent polarity plays a significant role in extracting flavones, such as baicalin, baicalein, wogonoside, and wogonin, from *S. baicalensis* roots. These flavonoids, as mentioned before, differ in polarity due to their glycosylation state. Solvent polarity influences the extraction efficiency by determining solubility and the interaction between flavones and the solvent. Polar solvents like water and high-concentration ethanol excel in extracting flavonoid glycosides (e.g., baicalin and wogonoside). This is because these compounds are hydrophilic and form hydrogen bonds with polar solvents. Aglycones, being less polar, have lower solubility in highly polar solvents. Mixtures of ethanol and water (e.g., 50–70% ethanol) are commonly used to extract both glycosides and aglycones effectively. The balance between polarity and hydrophobicity accommodates both compound types. On the other hand, non-polar solvents like hexane, dichloromethane, or chloroform are ideal for isolating aglycones like baicalein and wogonin, which have limited polarity. However, these solvents are ineffective for glycosides due to their hydrophilicity and significantly toxic for living plants.

**Figure 10:**
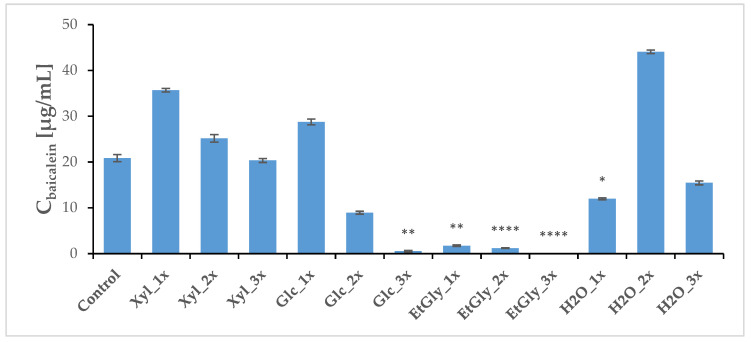
Average concentrations of main flavonoids in the *S. baicalensis* root extract—aglycone comparison, baicalein. Symbols correspond to statistically significant (**** *p* < 0.00005; ** *p* < 0.005 * *p* < 0.05) *t*-test results for independent groups. The error bars indicate the standard deviation (±SD).

**Figure 11:**
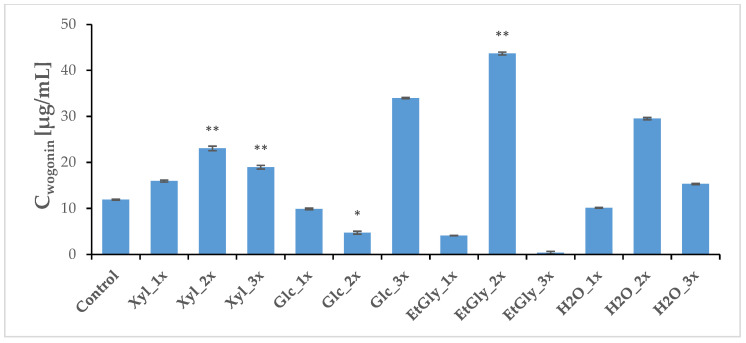
Average concentrations of main flavonoids in the *S. baicalensis* root extract—aglycone comparison, wogonin. Symbols correspond to statistically significant (** *p* < 0.005 * *p* < 0.05) *t*-test results for independent groups. The error bars indicate the standard deviation (±SD).

Across the board, the H_2_O_2x group emerged as a complete success—not only ensuring the aforementioned high PSR and root length increase (Table 4) but also strongly and consistently increasing the wogonin and baicalein content, 2.5-fold and 2.1-fold, respectively (Figure 10 and Figure 11, Table 5).

## 3. Materials and Methods

### 3.1. Plant Cultivation

**Figure 12:**
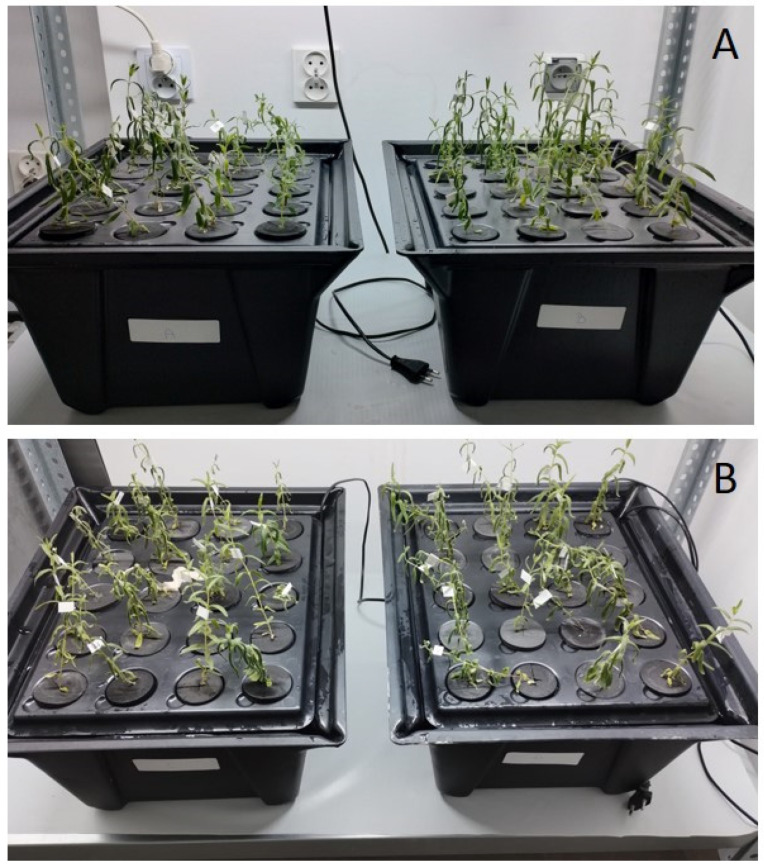
Six-week-old *S. baicalensis* cultivated in X-stream aero systems on the day of the experiment, right before the electroporation procedure (**A**), and three weeks after PEF treatment (**B**).

Seeds of *S. baicalensis* used for this experiment were acquired from the collection of the Botanical Garden of Medicinal Plants at the Medical University of Wrocław. Seeds were sterilized in NaClO (3% *w*/*v*) for 5 min, then rinsed 4 times with sterile water, stratified at 4 °C for four days, and then placed in a growth chamber at 22 °C, with a 16/8 h light/dark photoperiod. After sprouting, 115 seedlings (primary root length of approx. 2 cm) were moved to X-stream aero aeroponic systems (NutSystems Ltd., Farnham, UK) filled with water purified via reverse osmosis (Power Grow 500 reverse osmosis filter, Growmax Water, Salt Lake City, UT, USA). Following this, 20 mL (5 mL per 1 L of water) of ROOT!T First Feed (HydroGarden Ltd, Coventry, UK) nutritional medium (composition: 1.7% NO_3_, 0.1% NH_4_, 2.2% K_2_O, 1.0% P_2_O_5_, 1.9% CaO, 0.9% MgO, 0.6% SO_3_, 0.04% Fe, and 0.007% Zn) was then added and replenished every week. Plants were kept at 20–24 °C and exposed to white light for 8 h/day. To protect the plants from pests, the Lurem-TR biological system and Swirski Ulti-Mite (Koppert Biological Systems, Berkel en Rodenrijs, The Netherlands) sachets and sticky boards were used. Upon reaching three weeks of age, the roots of *S. baicalensis* seedlings (40 plants) were used for confocal microscopy imaging (Figure 1 and Figure 2). The remaining 75 specimens were kept under these growing conditions to reach six weeks of age (Figure 12A). On the experiment day, five of them were used to prepare methanolic extracts (extraction control, used for reference in comparing the yield of NADES extracts), 60 specimens were divided into 12 subgroups (5 specimens each, Table 1) treated with PEF, and the remaining 10 were sham-treated (survivability control, used as a reference in evaluating the impact of PEF treatment on plant fitness, Table 4). After PEF treatment, the plants were returned to the systems, and their position was randomized. They were observed for three more weeks (Figure 12B) and finally harvested in order to evaluate the changes the PEF caused in the *S. baicalensis* root phytochemical profile.

### 3.2. Confocal Microscopy Imaging

Roots of three-week-old *S. baicalensis* seedlings underwent PEF treatment of varying electric field strengths (1, 3, and 7.5 kV/cm), with other parameters constant (pulse width t = 50 μs, no. of pulses delivered N = 50, frequency f = 1 Hz) in either KCl or HEPES buffer mixed with 0.1 µM propidium iodide (PI). Each treatment was done on 10 plants, while the control consisted of 10 sham-treated plants. A BTX ECM 830 pulse generator (BTX, Waltham, MA, USA) and single-use cuvettes with a 4 mm gap between the electrodes were used in this process. Imaging was done on a Leica SP8 confocal microscope using an air 20× lens (NA 0.75). PI was excited with a 552 nm laser line, and the collected emission range was set at 560–680 nm. Six to eight areas were imaged with volumes up to 146 µm and a 2 µm Z step. For analysis, an optical section at 60 µm of depth starting from the first root layer was chosen (Figure 1). Images were analyzed using Fiji 2.12 [44] and ImageJ (ver. 1.54, National Institutes of Health, Bethesda, MD, USA) software. Nuclei were marked with a paintbrush tool in a separate empty channel, and upon channel binarization, they were counted with the Analyze Particle function. The graph (Figure 2) shows the number of nuclei per mm^2^ of the root section with means and standard deviations.

### 3.3. NADES Preparation

Natural Deep Eutectic Solvent mixtures (Table 1) used in this experiment as extractive–conductive media were prepared by continuously stirring the components of each mixture in a capped flask heated to 80 °C until a uniform, colorless liquid was formed in accordance with widely applied methods [40,41]. Choline chloride (≥99.0%, POL-AURA, Morąg, Poland) was used as a hydrogen bond donor, mixed with the following hydrogen bond acceptors: D-(+)-xylose (≥98.5% POL-AURA), glucose (≥99.0% pure PEPEES S. A., Łomża, Poland), and ethylene glycol (≥98.5% WARCHEM Sp. z.o.o., Warszawa, Poland). Water used in NADES preparation was purified by reverse osmosis (Power Grow 500 reverse osmosis filter).

### 3.4. PEF Treatment

**Figure 13:**
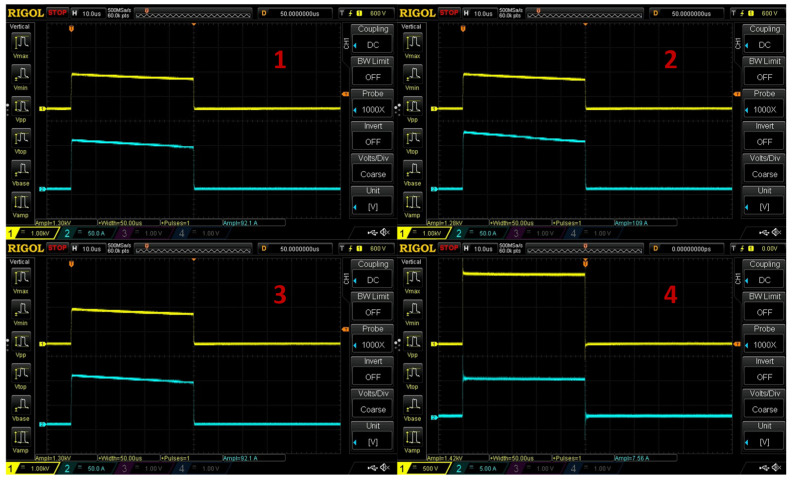
Oscilloscope images of delivered pulses in real time depending on the solvent used. ((**1**)—choline chloride/xylose (1:2) + 30% water, (**2**)—choline chloride/glucose (1:2) + 30% water, (**3**)—choline chloride/ethylene glycol (1:2), and (**4**)—tap water. The yellow line indicates recorded pulse intensity U [kV], and the blue line, the impedance [A]. The differential probe was set at 2000×, and the oscilloscope recording was magnified by 1000×, meaning that the real U values were twice the recorded amount.

Six-week-old roots of *S. baicalensis* were electroporated in freshly prepared, room-temperature NADES solvents (5 plants per treated subgroup). PEF treatment of the following parameters was used: E = 0.3 kV/cm (electric field intensity U = 3 kV, distance between electrodes d = 10 cm), t = 50 µs, N = 33 f = 1 Hz. This treatment was carried out in cuvettes with a movement distance range of a 1 to 10 cm gap between the electrodes. PEF treatment was either applied in this process once, or repeated two or three times with a five-minute resting period after each electroporation (e.g., Glc_1x—plants in Glc NADES mixture, PEF applied once, Glc_2x—plants in Glc NADES mixture, PEF applied twice, Glc_3x—plants in Glc NADES mixture, PEF applied three times). A 10 kV pulse generator (VITAVE, Prague, Czech Republic) was used to generate the impulses, and 100 × 53 × 2.5 mm copper silver-coated electrodes were used to transfer the current. The pulse parameters were monitored in real time (Figure 13) using a CT4068-NA differential probe (Cal Test Electronics, Yorba Linda, CA, USA) and recorded using a Rigol DS1054Z oscilloscope (Rigol Technologies, Co., Ltd., Suzhou, China). Temperature and conductivity measurements of NADES electroporation media were carried out using HI98304 DiST 4 EC-meter (Hanna Instruments, Smithfield, RI, USA) before and after each PEF application. Total specific energy input (Wspec) was calculated based on recorded measurements (E, t, and N recorded on the oscilloscope, EC recorded on the EC-meter, combined sample mass of medium and treated plant weighed on a scale) as reported in other works in this field [35,36].

Depending on how many times the treatment was repeated and which substance was used as an extractive–conductive medium, the experiment was divided into subgroups (Table 1). The control group (10 plants) was subjected to the sham treatment (except the PEF application) in the same conditions and with the maximum time spent in the cuvette (15 min). Then, plants were transferred to a beaker with reverse osmosis-purified water, containing a magnetic stir bar, and placed on a magnetic stirrer for 15 min. This ensured constant agitation, thoroughly washing the roots to remove sugars and prevent contamination. Subsequently, plants were returned to the systems, and their positions were randomized (randomly pairing plant numbers with slot numbers in the system’s lid) to homogenize cultivation conditions after the treatment (Figure 12). Their growth and development were then monitored for three weeks. Weekly photographic documentation on a gridline allowed for the measurement of root and shoot length increases using ImageJ software (National Institutes of Health, Bethesda, MD, USA).

### 3.5. Preparation of Extracts for HPLC-MS Analysis

After the PEF application was carried out, the NADES mixtures were collected. Subsequently, they were purified using the Solid Phase Extraction (SPE) technique on a Vantage L Laboratory Column (VL 22 × 250, Millipore, Burlington, MA, USA) filled with 140 nm pore size octadecylsilane stationary phase. The column was conditioned using 100% analytical-grade methanol and then washed with distilled water. NADES extract was then introduced, followed by washing with distilled water to elute the sugars first. Finally, 80% analytical-grade methanol was used to elute all compounds of interest from the extract. Control extracts (extraction control to be compared to NADES in order to evaluate their extraction efficiency) were prepared by harvesting 5 intact plants on the day of the experiment, drying (Klarstein Pro Master Jerky 300 dehydrator, T = 35–40 °C) and separating their roots, grinding them, and conducting regular ultrasound-mediated (Intersonic IS-27, Olsztyn, Poland) extraction, using 80% analytical-grade methanol as a solvent. The program was set to 20 s of delivering ultrasounds and a 5 s pause at 25 °C for 30 min. Afterwards, the samples were centrifuged (5000 rpm for 5 min, Hermle Labortechnik GmbH, Z 206 A centrifuge, Wehingen, Germany) and supernatants were collected. This process was repeated three times. After the three-week observation period, all plants (60 from the treated groups and 10 from the sham-treated control group) were subjected to extraction in the same manner as the extraction control group. All final extracts were then dried in a rotary evaporator (Rotavapor R-100V, BÜCHI Labortechnik AG, Flawil, Switzerland). One mg of each of the residues was subsequently dissolved in 1 mL of HPLC-grade 80% methanol with 0.1% formic acid and purified using 0.20 µm filters (Chromafil PTFE disposable syringe filters, Macherey-Nagel GmbH & Co., Düren, Germany). Each of the purified extracts was then divided into three 0.2 mL samples for HPLC-MS analysis.

### 3.6. HPLC-MS Analysis

All *S. baicalensis* extract samples were analyzed by UHRMS on a Dionex UltiMate 3000RS (Thermo Scientific, Darmstadt, Germany) system interfaced with a high-resolution quadrupole time-of-flight mass spectrometer (HR/Q-TOF/MS, Impact II, Bruker Daltonik GmbH, Bremen, Germany). *S. baicalensis* metabolome (Table S6) was chromatographically separated on an Acquity UPLC BEH C18 column (100 × 2.1 mm, 1.7 μm, Waters, Manchester, UK) maintained at 30 °C. The mobile phase consisted of A (0.1% formic acid in Milli-Q water, *v*/*v*) and B (0.1% formic acid in acetonitrile, *v*/*v*) at a flow rate of 0.4 mL/min. The gradient elution was 2% B from 0 to 1 min with a short 0.3 min calibration segment, and the concentration of B was then increased to 60% from 1 to 20 min. The column was eluted with this concentration of solvent B for 4 min and then re-equilibrated for 0.3 min and back to 2% of B for the next 3.7 min. The samples were kept at 15 °C in the autosampler. The injection volume was 5.0 μL. The mass spectrometer operated in the positive and negative electrospray ionization ESI mode. The ion source operated with the following parameters: capillary voltage of 4.0 kV, corona voltage of 8.0 kV, nebulizer pressure of 2.5 bar, dry gas flow of 1.5 L/min, dry temperature of 200 °C, and vaporizer temperature of 320 °C. The mass scan range was from 50 to 1200 *m*/*z* with a 5 Hz spectral acquisition rate. MS/MS spectra were acquired in a data-dependent manner, whereby precursor ions (maximum 2) from each scan were subjected to collision-induced fragmentation if their absolute intensity exceeded 1800 counts. Variable collision energy ranging from 15 to 35 eV was used depending on the *m*/*z* of the selected precursor ion. Internal calibration was performed with sodium formate and introduced to the ion source via a 20 μL loop at the beginning of each analysis using a six-port valve. Data were collected and processed by DataAnalysis 4.3 (Bruker Daltonik GmbH, Bremen, Germany). All analyses were performed in triplicate. Baicalein, baicalin, wogonin, wogonoside, oroxylin A, and oroxyline A-7-glucuronide were purchased for standards stock solutions from Sigma-Aldrich (St. Louis, MO, USA), kept frozen until analysis, and prepared by dissolution in 80% LC-MS grade MeOH, resulting in range concentrations for each standard of 1.1, 0.9, 1.4, 1.1, 1.3, and 0.9 mg/mL, respectively. Such concentrations were then used to prepare the calibration curves based on seven concentration points (from 800 to 14 µg/mL), and a UV chromatogram was recorded at 280 nm. Obtained MS spectra were automatically exported to a MetaboScape 2021 (Bruker, Daltonik GmbH, Bremen, Germany). The retention time range was set between 2 and 22 min, and the mass range was between 100 and 800 *m*/*z*. The compound concentrations were calculated from the standard curve produced by plotting the peak area of standard solutions (Table 2) of respective compounds to their known concentrations. The significance of differences in concentrations of compounds was assessed by statistical tests, e.g., the *t*-test for independent groups or two-way ANOVA with Tukey’s multiple comparison test (95% probability level) (Table S7).

### 3.7. Materials

A 3% solution of sodium hypochlorate (NaClO) used for seed purification was purchased from Chempur Sp. z o. o. (Piekary Śląskie, Poland). ROOT!T First Feed (HydroGarden Ltd, Coventry, UK) was used as a nutritional medium for the plants.

The following substances were used in NADES preparation: choline chloride (≥99.0%, POL-AURA, Oława, Poland), D-(+)-xylose (≥98.5% POL-AURA), glucose (≥99.0% pure PEPEES S. A., Łomża, Poland), and ethylene glycol (≥98.5% WARCHEM Sp. z.o.o., Zakręt, Poland). Analytical-grade methanol (Chempur Sp. z o. o., Piekary Śląskie, Poland) and distilled water (DE 10 electric distillation unit, Polna, Przemyśl, Poland) were used for SPE separation, where 140 nm pore size octadecylsilane (Cosmosil, Nacalai Tesque, Kyoto, Japan) served as a stationary phase. Ultrapure distilled water (Milli-Q, Darmstadt, Germany), LC-MS-grade formic acid (Carl Roth GmbH, Karlsruhe, Germany), and LC-MS-grade methanol (Merck KGaA, Darmstadt, Germany) were used for HPLC-MS sample preparation. During HPLC-MS analysis, the same Milli-Q water and LC-MS-grade acetonitrile (Merck KGaA, Darmstadt, Germany) were used as mobile phases. Propidium iodide used for nuclei staining was purchased from Thermo Scientific (Waltham, MA, USA). Potassium chloride (Chempur Sp. z o. o., Piekary Śląskie, Poland) solution in distilled water (DE 10 distillation unit) and HEPES buffer (Sigma-Aldrich, St. Louis, MO, USA) were used as electroporation media in this experiment.

### 3.8. Statistical Analysis

To evaluate data quality and trends, several statistical analyses were performed using GraphPad Prism version 5.01 (GraphPad Software, LLC., San Diego, CA, USA). The Mann–Whitney U test, Kendall tau correlation, and Pearson’s correlation tests were used to assess the impact of PEF application and treatment intensity on the concentrations of compounds in the NADES extracts (Tables S3–S5). A *t*-test for independent groups was employed to determine statistically significant differences in plant growth parameters (e.g., root length increase, fresh mass, Table 4) and the concentrations of key flavonoids in the NADES extracts and to analyze changes in the *S. baicalensis* root phytochemical profile after harvest (Table 3 and Table 5). A two-way ANOVA (95% probability level) with Tukey’s post hoc test was used to analyze confocal microscopy data (Table S2) and identify differences in the *S. baicalensis* metabolome caused by PEF treatment in comparison to the sham-treated control (Table S7). Additionally, multivariate principal component analyses were performed. Rectangular bucketing with parameters acquired from time alignment was used. Data were normalized by the sum of bucket values in analysis and Pareto scaling; afterwards, PCA and OPLS-DA were conducted (Figures S1–S4). The value count of buckets was set to be equal to or greater than 25%.

## 4. Conclusions

The Xyl_2x treatment proved to be the most effective for flavonoid extraction from living *S. baicalensis* plants, a process that could be referred to as “root milking.” In this case, the yield of wogonin was equivalent to that achieved through conventional methanolic extraction (Figure 6). Moreover, the three-week plant survival rate (PSR) of plants from this subgroup was 60%, which allows for potential repeatability of the extraction process, contrary to the control group, where all plants perished in order to prepare the extract.

The phytochemical profile of *S. baicalensis* was significantly influenced by the application of PEF, especially in terms of aglycone content. This suggests that PEF is a promising method for the targeted extraction of pharmacologically active aglycones. Our findings indicate that the extraction of aglycones is not only dependent on the intensity of the electric field but also on the conductivity of the extraction medium. Among the tested solvents, NADESs showed better performance compared to water, which has a lower conductivity. Glucose, in particular, contributed to higher conductivity due to its molecular interactions, resulting in increased solvent viscosity and better mobility of ionic species.

PEF treatment also promoted plant growth, especially with low-intensity treatments, likely due to the mild stress induced by the electric field. The increase in biomass is a very desirable outcome and could contribute to higher compound yields over time.

In summary, PEF treatment paired with biocompatible solvents is a promising non-lethal method for extracting active metabolites, although further optimization is necessary. Although more bioactive aglycones such as baicalein and wogonin were more successfully extracted in this study, glycosides like baicalin and wogonoside are much more stable, making them more suitable for pharmaceutical formulations that require longer shelf lives. The main challenge we face is the fine-tuning of PEF parameters, such as electric field strength (E), pulse width (t), pulse number (N), and frequency (f), to optimize the yield of both aglycones and glycosides, while minimizing potential cell damage. Firstly, this study will be expanded upon by testing the same treatment conditions on at least 100 one-year-old *S. baicalensis* plants in order to improve the phytochemical content of the NADES extracts and yield more valuable data for statistical analysis. This project would also involve applying PEF to the roots multiple times throughout the year and comparing the final yield with that of traditional methanolic extraction.

Increasing extraction yield while maintaining plant survival is a vital aim of our research endeavors. Future perspectives for this approach include investigating a broader range of NADES solvents’ (e.g., lactic acid with glucose or fructose with urea) potential as conductive–extractive media, as well as combining PEF treatment with ultrasound in order to further facilitate metabolite extraction, with attention given to solvent polarity and the mechanical disruption of root tissues. Additionally, the effect of temperature on extraction efficiency will be evaluated at various temperatures (25–40 °C) to understand the thermal impact on solubility and compound stability. These efforts will result in the development of a predictive model, combining solvent polarity, pH, temperature, and extraction techniques in order to optimize flavonoid yields. Future studies will also investigate other plants producing valuable compounds, as well as alternative solvents like ionic liquids or liquid CO_2_ for environmentally sustainable extractions. These efforts will help optimize the extraction of bioactive compounds for use in nutraceutical, pharmaceutical, and cosmetic applications. We hope that in the future, our efforts could pave the way for integrating this method into sustainable production pipelines for active metabolites at an industrial scale.

## Figures and Tables

**Table 1:** Treated subgroups description. The symbols 1×, 2×, and 3× indicate how many times the treatments were applied. Electrical conductivity (EC) measurements were taken at room temperature (T = 23–25 °C).

Subgroup Name	Solvent Used, NADES Media (1–4)
Xyl_1x	1—choline chloride/xylose (1:2) + 30% water EC = 7.97 mS/cm
Xyl_2x	
Xyl_3x	
Glc_1x	2—choline chloride/glucose (1:2) + 30% water EC = 9.72 mS/cm
Glc_2x	
Glc_3x	
EtGly_1x	3—choline chloride/ethylene glycol (1:2) EC = 8.61 mS/cm
EtGly_2x	
EtGly_3x	
H_2_O_1x	4—tap water EC = 0.7 mS/cm
H_2_O_2x	
H_2_O_3x	

**Table 2:** Main flavonoids identified in the control MeOH extract of six-week-old *S. baicalensis*. ^a^ Compounds extracted for calibration curve equations and limits of detection (LOD) and quantification (LOQ) for flavonoids.

No.	Compound Name	Rt [min]	UV	*m*/*z* [M − H]-	Error [ppm]	Formula	MS^2^ Ion	*m*/*z* [M + H]+	MS^2^ Ion	LOQ [µg/mL]	LOD [µg/mL]
1	6-C-Arabinose-8-C-glucose-chrysin	9.7	215, 275, 315	547.1447	0.1	C_26_H_28_O_13_	337 (100), 367 (83), 457 (13), 309 (12), 427 (10)	549.1602	309 (100), 375 (94), 321 (90), 279 (72)		
2	6-C-Glucose-8-C-arabinose-chrysin	10.2	215, 275, 315	547.1446	0.1	C_26_H_28_O_13_	337 (100), 367 (68), 427 (44), 457 (27)	549.1597	351 (100), 271 (35)		
3	Scutellarein-7-O-β-D-glucuronide-5-glucoside	10.4	284, 333	623.1987	0.3	C_29_H_36_O_15_	461 (50), 285 (24)	625.1997	161 (80)		
4	Scutellarein glucoside	11.4	210, 270	447.0935	0.1	C_21_H_20_O_11_	285 (100)	449.1459	287 (100)		
5	Baicalin ^a^	12.6	225, 280, 315	445.0771	0.3	C_21_H_18_O_11_	269 (100), 251 (8)	447.0922	271 (100), 253 (2)	0.55	0.18
6	Oroxylin A 7-O-β-D-glucuronide ^a^	13.8	210, 270, 310	459.0928	−1.2	C_22_H_22_O_11_	268 (100), 283 (16), 269 (13)	461.1084	270 (100), 285 (73)	0.41	0.37
7	Chrysin-glucuronide	13.9	270, 312	429.0821	0.3	C_21_H_18_O_10_	253 (100)	431.0975	255 (100)		
8	Wogonoside ^a^	14.4	220, 275	459.0929	0.9	C_22_H_22_O_11_	268 (100), 283 (15), 269 (13), 284 (2)	461.1085	270 (100), 285 (85)	0.41	0.17
9	Baicalein ^a^	14.8	215, 275, 320	269.0457	1.1	C_15_H_10_O_5_	223 (24), 241 (20), 195 (17), 169 (15), 197 (12), 251 (11), 271 (8)	271.0603	123 (79), 253 (21), 169 (20), 103 (8)	0.45	0.16
10	Norwogonin	16.4	220, 280	269.0453	0.4	C_15_H_10_O_5_	197 (100), 171 (40), 213 (31)	271.0604	169 (100), 139 (12), 123 (9), 141 (8)		
11	Wogonin ^a^	19.1	210, 275	283.0616	0.3	C_16_H_12_O_5_	268 (100), 163 (23), 184,239	285.0753	270 (100), 179 (8), 252 (7)	0.62	0.2
12	Oroxylin A ^a^	19.5	215, 270, 320	283.0613	−0.5	C_16_H_12_O_5_	268 (100), 165 (9), 184 (7), 240	285.0754	270 (100), 168 (46), 140 (9), 242 (8), 224 (3)	0.72	0.22

**Table 3:** Average concentrations of wogonoside and wogonin across extracts. Symbols correspond to statistically significant (**** *p* < 0.00005; *** *p* < 0.0005 * *p* < 0.05) *t*-test results for independent groups. SD—standard deviation (n = 3).

Group	Wogonoside [μg/mL]	SD	Wogonin [μg/mL]	SD
Control	41.02	0.34	2.15	0.01
Xyl_1x	1.64 ****	0.12	0.25 *	0.02
Xyl_2x	3.01 ****	0.60	2.14	0.01
Xyl_3x	4.50 ****	0.99	1.35 ***	0.02
Glc_1x	1.42 ****	0.51	0.30 ****	0.10
Glc_2x	1.35 ****	0.12	0.47	0.14
Glc_3x	1.62 ****	0.86	0.47	0.15
EtGly_1x	3.13 ****	0.05	ND ^1^	
EtGly_2x	1.02 ****	0.001	ND	
EtGly_3x	1.34 ****	0.008	ND	
H2O_1x	0.23 ****	0.0009	ND	
H2O_2x	1.94 ****	0.03	ND	
H2O_3x	0.93 ****	0.10	ND	

^1^ ND—not determined.

**Table 4:** Average (Avg) root and shoot growth, root dry and wet mass, plant survival rate (PSR), and plant growth and development (PGD) observed over a three-week period after PEF treatment. Symbols correspond to statistically significant (** *p* < 0.005 * *p* < 0.05) *t*-test results for independent groups. SD—standard deviation (n = 3).

Group	Avg Root Length Increase [cm]	SD	Avg Root Wet Mass [g]	SD	Avg Root Dry Mass [g]	SD	Avg Shoot Length Increase [cm]	SD	PSR	PGD
Control	9.41	5.16	0.41	0.19	0.11	0.03	5.61	1.92	100%	80%
Xyl_1x	13.59	4.00	0.70	0.33	0.13	0.04	3.56 *	0.85	80%	60%
Xyl_2x	9.20	0.78	0.71	0.45	0.17	0.06	2.33 **	0.39	60%	60%
Xyl_3x	6.97	3.89	0.59	0.41	0.13	0.05	2.95 *	1.21	60%	40%
Glc_1x	9.28	2.03	0.68	0.39	0.15	0.03	2.38 *	0.87	80%	60%
Glc_2x	8.12	4.47	0.32	0.19	0.11	0.03	2.25 **	0.31	80%	40%
Glc_3x	7.03	2.06	0.30	0.16	0.08 *	0.04	2.14 **	0.51	60%	20%
EtGly_1x	6.83	2.63	0.40	0.21	0.11	0.02	1.97 **	0.32	60%	60%
EtGly_2x	6.04	0.93	0.24	0.13	0.09 *	0.03	4.01 *	0.68	60%	20%
EtGly_3x	4.41 *	0.98	0.39	0.28	0.09 *	0.03	2.11	1.24	60%	20%
H2O_1x	11.24	6.49	0.74	0.57	0.15	0.09	7.03	2.11	60%	60%
H2O_2x	13.47	3.24	0.56	0.39	0.12	0.05	3.93	0.63	60%	60%
H2O_3x	13.87 *	1.76	0.88 *	0.41	0.17*	0.04	6.20	1.31	80%	80%

**Table 5:** Average concentration of main flavonoids in *S. baicalensis* roots harvested three weeks after PEF treatment (**** *p* < 0.00005; ** *p* < 0.005 * *p* < 0.05 *t*-test results for independent groups). SD—standard deviation (n = 3).

Group	Wogonoside [μg/mL]	SD	Baicalin [μg/mL]	SD	Wogonin [μg/mL]	SD	Baicalein [μg/mL]	SD
Control	4480.56	50.03	594.57	15.52	11.91	0.10	20.86	0.77
Xyl_1x	4269.87	51.88	635.04	12.95	15.96	0.22	35.69	0.38
Xyl_2x	3695.17	75.42	520.95	9.87	23.05 **	0.50	25.17	0.83
Xyl_3x	2991.40	51.77	381.86 *	4.56	18.97 **	0.40	20.33	0.43
Glc_1x	5520.29 **	258.41	635.14	39.40	9.91	0.18	28.75	0.63
Glc_2x	2992.32 *	14.61	475.34 **	3.65	4.73 *	0.31	8.94	0.32
Glc_3x	832.81 **	11.98	115.83 ****	1.51	33.98	0.15	0.54 **	0.16
EtGly_1x	1617.56 *	20.53	236.94 **	2.96	4.11	0.04	1.73 **	0.15
EtGly_2x	387.01 ****	3.20	54.84 ****	0.39	43.68 **	0.33	1.21 ****	0.05
EtGly_3x	4.18 ****	0.09	0.29 ****	0.04	0.37	0.29	0.02 ****	0.01
H2O_1x	3780.64	33.06	552.01	12.19	10.14	0.07	11.98 *	0.20
H2O_2x	4124.55	32.43	609.29	7.90	29.53	0.26	44.07	0.39
H2O_3x	3444.95	28.09	546.07	8.69	15.32	0.13	15.45	0.43

Group

Wogonoside [μg/mL]

SD

Baicalin [μg/mL]

SD

Wogonin [μg/mL]

SD

Baicalein [μg/mL]

SD

## Data Availability

The experimental data are available upon request at sylwester.slusarczyk@umw.edu.pl.

## Supplementary Materials

The supporting information can be downloaded at https://www.mdpi.com/article/10.3390/ijms26010100/s1.
