# Supplementary material for: Pulsed Electric Field (PEF) Treatment Results in Growth Promotion, Main Flavonoids Extraction, and Phytochemical Profile Modulation of Scutellaria baicalensis Georgi Roots
Source: Int J Mol Sci. 2024 Dec 26;26(1):100. doi: 10.3390/ijms26010100 (PMC11719946; doi:10.3390/ijms26010100)
Supplement: Supplementary file 1 [file ijms-26-00100-s001.zip › ijms-3355684-supplementary.pdf]

International Journal of
Molecular Sciences

MDPI

Supplementary

Pulsed Electric Field (PEF) treatment results in growth promotion, main flavonoid extraction, and phytochemical profile modulation of *Scutellaria baicalensis* Georgi roots

Kajetan Grzelka [1], Adam Matkowski [1,2], Grzegorz Chodaczek[3], Joanna Jaśpińska[2], Anna Pawlikowska-Bartosz[2], Wojciech Słupski[4], Dorota Lechniak[5], Małgorzata Szumacher-Strabel[6], Segun Olorunlowu[7], Adam Cieślak[8,*] and Sylwester Ślusarczyk [2,*]

* **Correspondence:** Sylwester Ślusarczyk: sylwester.slusarczyk@umw.edu.pl

Table S1. Detailed data regarding confocal microscopy. PI was excited with a 552 nm laser line and the collected emission range was 560-680 nm. Six to eight areas were imaged with volumes up to 146 um and a 2 um Z step. For analysis, an optical section at 60 um of depth starting from the first root layer was chosen.

Copyright: © 2024 by the authors. Licensee MDPI, Basel, Switzerland. This article is an open access article distributed under the terms and conditions of the Creative Commons Attribution (CC BY) license (https://creativecommons.org/licenses/by/4.0/).

Conditions	Series	Layer	No. of nuclei	Layer area (pixels)	Layer area [µm²]	No. of nuclei [µm²]	No. of nuclei [mm²]
1000 V	s036	34	16	616068	127038.2913	0.000125946	125.946278
	s037	35	11	606701	125106.7388	8.79249E-05	87.92492
	s038	35	9	586638	120969.5831	7.43989E-05	74.3988676
	s039	35	15	598901	123498.3146	0.000121459	121.459147
	s040	36	3	690596	142406.5782	2.10664E-05	21.0664426
	s041	37	36	612322	126265.8352	0.000285113	285.112754
	s042	36	14	730474	150629.7499	9.29431E-05	92.9431272
	s043	35	18	729105	150347.4509	0.000119723	119.722682
3000 V	s044	40	10	499300	102959.7688	9.71253E-05	97.1253152
	s045	35	35	560130	115503.4153	0.000303021	303.021343
	s046	35	13	662657	136645.3264	9.51368E-05	95.1368066
	s047	35	9	677309	139666.6894	6.44391E-05	64.4391303
	s048	37	20	619123	127668.2574	0.000156656	156.656012
	s049	34	23	734826	151527.1681	0.000151788	151.787962
	s050	34	49	720372	148546.6344	0.000329863	329.862741
	s051	34	100	757818	156268.3077	0.000639925	639.92502
7500 V	s052	37	98	572168	117985.75	0.000830609	830.608781
	s053	36	82	584728	120575.7254	0.000680071	680.070551
	s054	36	39	701310	144615.8931	0.00026968	269.679903

	s055	34	41	766437	158045.6165	0.000259419	259.418774
	s056	36	48	505062	104147.9406	0.000460883	460.882853
	s057	36	45	518596	106938.7628	0.000420802	420.801577
	s058	36	59	571766	117902.8543	0.000500412	500.411973
	s059	36	77	567618	117047.5026	0.000657853	657.852567
NO electroporation,	s060	35	9	659137	135919.4735	6.62157E-05	66.2156773
PI present	s061	38	11	679252	140067.352	7.85336E-05	78.5336472
	s062	36	38	835795	172347.8068	0.000220484	220.484384
	s063	34	16	767337	158231.2039	0.000101118	101.117855
	s064	36	5	743773	153372.1132	3.26005E-05	32.6004506
	s065	36	14	649786	133991.2203	0.000104484	104.484458
	s066	37	15	572334	118019.9806	0.000127097	127.097123
	s067	35	3	567217	116964.8131	2.56487E-05	25.6487393
NO electroporation,	s068	-	0		0	N/A	N/A
NO PI	s069	39	0		0	N/A	N/A
	s070	36	0		0	N/A	N/A
1000 V hepes	s090	36	39	590949	121858.5468	0.000320043	320.043206
	s091	36	32	650623	134163.8166	0.000238514	238.514383
	s092	34	21	671068	138379.7439	0.000151756	151.756315
	s093	34	16	714798	147397.2297	0.00010855	108.550208
	s094	35	18	746594	153953.8266	0.000116918	116.918172
	s095	35	37	671568	138482.848	0.000267181	267.181103
3000 V hepes	s096	36	28	831971	171559.2665	0.000163209	163.208905
	s097	35	33	685245	141303.1579	0.00023354	233.540428
	s098	36	8	675706	139336.1376	5.74151E-05	57.4151124
	s099	36	14	776949	160213.2774	8.73835E-05	87.3835192
	s100	36	15	814493	167955.1591	8.93096E-05	89.3095519
	s101	36	31	593346	122352.8279	0.000253366	253.365619
7500 V hepes	s102	35	48	786661	162215.9717	0.000295902	295.901812
	s103	35	90	740554	152708.3289	0.000589359	589.358817
	s104	35	169	781943	161243.0813	0.001048107	1048.10699
	s105	36	71	741468	152896.8032	0.000464365	464.365497
	s106	34	86	789592	162820.368	0.000528189	528.189446
	s107	34	71	859719	177281.1325	0.000400494	400.493831

Table S2. Confocal imaging multiple comparisons test results for 0, 1000, 3000 and 7500 V/cm ANOVA with Tukey's multiple comparison test (**** $p < 0.00005$; *** $p < 0.0005$ ** $p < 0.005$ * $p < 0.05$, ns – not significant).

Calculator	0.20620823
No. of pixels in a photo	1048576
Photo area [μm^2]	216225

**ANOVA with Tukey's multiple comparisons test**

0 V vs. 1000 V	ns
0 V vs. 3000 V	ns
0 V vs. 7500 V	****
0 V vs. 1000 V hepes	ns
0 V vs. 3000 V hepes	ns
0 V vs. 7500 V hepes	****
1000 V vs. 3000 V	ns
1000 V vs. 7500 V	***
1000 V vs. 1000 V hepes	ns
1000 V vs. 3000 V hepes	ns
1000 V vs. 7500 V hepes	****
3000 V vs. 7500 V	*
3000 V vs. 1000 V hepes	ns
3000 V vs. 3000 V hepes	ns
3000 V vs. 7500 V hepes	**
7500 V vs. 1000 V hepes	**
7500 V vs. 3000 V hepes	**
7500 V vs. 7500 V hepes	ns
1000 V hepes vs. 3000 V hepes	ns
1000 V hepes vs. 7500 V hepes	**
3000 V hepes vs. 7500 V hepes	***

**Table S3. U Mann-Whitney test results. Continuous variable: PEF treatment (equals 0 in sham-treated Control and 1 in Treated). Highlighted p values are significant (<0.05). N – no. of measurements of concentration of given compound taken form analysed**	**Rank sum. Treated**	**Rank sum. Control**	**U**	**Z**	**p**	**N Treated**	**N Control**	**Two-tailed exact test (p-value)**

**samples.Avg C [µg/mL]**								
$C_{baicalein}$	1957.0	254.0	127.0	-1.1710	0.2416	60	6	0.2485
$C_{baicalin}$	1912.0	299.0	82.0	-2.1747	0.0297	60	6	0.0269
$C_{wogonoside}$	1860.0	351.0	30.0	-3.3346	0.0009	60	6	0.0002
$C_{wogonin}$	2040.0	171.0	150.0	0.6580	0.5105	60	6	0.5199

Table S4. Kendall tau correlation results. Highlighted p values are significant (<0.05). N – no. of pairs.

**Variable pair**	**N**	**Kendall's Tau**	**Z**	**p**
Wspec [kJ/kg] & $C_{wogonoside}$	36	0.2335	2.0039	0.0451
Wspec [kJ/kg] & $C_{wogonin}$	18	0.2622	1.5201	0.1285

Table S5. Pearson's correlation results. Data normalization was carried out using Box-Cox transformation. Highlighted p values are significant (<0.05).

**Variable**	**$C_{wogonoside}$ normalized**	**$C_{wogonin}$ normalized**
Wspec normalized	p = 0.4477	p = 0.4909

Table S6. *Scutellaria baicalensis* Georgi root phytochemical profile. Identification based on mass spectrometer operated in the negative and positive electrospray ionization ESI mode compared with isolated standards[a] or tentatively identification with CompoundCrowler 3,1 program (Bruker Daltonoics Darmstad), supported with MetFrag database and appropriate literature.

No.	Compound [Reference]	Rt[min]	UV λ max nm	m/z[M-H]$^-$	Formula	MS^2 ion	m/z[M+H]$^+$	MS^2 ion
1	tryptophan	1.8	220, 280	203.0000	$C_{11}H_{12}N_2O_2$	116(100), 142(47)	205.0971	188(100), 146(57)
2	unknown	2.6	220, 277	431.1561	$C_{19}H_{28}O_{11}$	191(100), 119, 299, 237	433.1704	455(M-Na)
3	taxifolin-3-glucopyranoside	3.1	288	465.1049	$C_{21}H_{22}O_{12}$	339(100), 285(74), 303(12), 125(100)		
4	taxifolin-7-glucopyranoside	4.6	290	465.1041	$C_{21}H_{22}O_{12}$	303(100)	467.1185	489[M+Na], 305(100), 287
5	quercetin-7-glucoside	5.0	253	463.0879	$C_{21}H_{20}O_{12}$	301(100), 285(41), 177, 125, 151		
6	unknown	5.1		447.1501	$C_{19}H_{28}O_{12}$	269(100), 401(24), 161, 121		
7	scuteamoenoside	5.2		463.1239	$C_{22}H_{24}O_{11}$	301(100), 139, 191, 161		
8	unknown rhamnosyl	5.4		475.1824	$C_{21}H_{32}O_{12}$	113(100), 149, 329(54)	477.1962	
9	unknown	5.6		401.1451	$C_{18}H_{26}O_{10}$	161(100), 269(75), 113(28)		
10	taxifolin	6.9	210, 290	303.0510	$C_{15}H_{12}O_7$	177(100), 125(58), 149(24)	305.0653	

11	unknown	7.3		683.1476	$C_{29}H_{32}O_{19}$	345(100), 330(41), 507(11)		
12	5,7,3,2'6'-pentahydroxy flavanone	7.5	210, 290	303.0510	$C_{15}H_{12}O_{7}$	177(100), 125(58), 149(24)	305.0652	
13	luteolin 7-O-β-D-glucoside	7.9		447.0944	$C_{21}H_{20}O_{11}$	285(100)		
14	taxifolin-glucuronide	8.0		479.0838	$C_{21}H_{20}O_{13}$	303(100), 285(17), 166(40)		
15	salviaflaside	8.2		521.1308	$C_{24}H_{26}O_{13}$	359(100)		
16	apigenin 7-O-[β-D-apiosyl-(1->2)-β-D-glucoside]	8.3	220, 291, 335	563.1397	$C_{26}H_{28}O_{14}$	239(100), 209(39), 443, 353, 269	565.1547	325(100), 379(89), 337(84)
17	5,7,3,2',6'-pentahydroxy flavanone-glucosyl (taxifolin 3-rhamnoside)	8.5	230, 291	449.1079	$C_{21}H_{22}O_{11}$	125(100), 177, 287	451.00	
18	dihydroapigenin 7-O-[β-D-apiosyl-(1→2)-β-D-glucoside]	8.7	220, 291, 335	565.1578	$C_{26}H_{30}O_{14}$	239(100), 209(72), 269(51)		
19	5,7,3,2',6'-pentahydroxy flavone	8.75		301.0345	$C_{15}H_{10}O_{7}$	149(100), 151(65), 175(25), 125(4)	303.0499	153(100), 179(50), 207(48), 229, 137
20	6-C-glucose-8-C-rhamnose-chrysin	8.8	220, 273, 315	577.1552	$C_{27}H_{30}O_{14}$	337(100), 457(75), 367(59), 309(13)	579.1709	309(100), 279(60), 363(54), 333(35)
21	eriodictyol-7-O-glucoside	9.2		449.1098	$C_{21}H_{22}O_{11}$	287(82), 161(37), 125		
22	5,7,3,2',6'-pentahydroxy flavanone(taxifolin)	9.3	210, 290	303.0510	$C_{15}H_{12}O_{7}$	177(100), 125(58), 149(24)	305.0653	
23	6-C-arabinose-8-C-glucose-glucose-chrysin	9.32		709.1960	$C_{32}H_{38}O_{18}$	443(100), 243	711.2122	549(80), 363(100), 375(90), 393(80), 321, 309
24	dihydroapigenin glucuronide	9.35		447.0938	$C_{21}H_{20}O_{11}$	271(100)		
25	dihydroscutellarein-7-O-β-D-glucuronide (eriodictyol 7-glucuronide)	9.4	215, 275	463.0895	$C_{21}H_{20}O_{12}$	287(100), 166(30), 181(15)	465.1024	289(100), 271(45), 169(24)
26	scutellarein-7-O-β-D-glucuronide[a]	9.5	220, 280, 330	461.0712	$C_{21}H_{18}O_{12}$	285	463.0870	287
27	6-C-Glucose-8-C-arabinose-chrysin	9.6	215, 275, 315	547.1448	$C_{26}H_{28}O_{13}$	337(100), 367(68), 427(44), 457(27)	549.1597	351(100), 271(35), 179, 167
28	5,7,2'-trihydroxy-6-methoxy flavone-7-O-β-D-glucuronide	9.7	215, 270, 337	475.0873	$C_{22}H_{20}O_{12}$	284(100), 299(35), 285(13.7), 300(5), 165(2.6)	477.1025	286(100), 301(73), 183(6), 168(5.5)
29	4',5,7-trihydroxy-flavone(apigenin)-glucuronide-glucose	9.8	274	607.1323	$C_{27}H_{28}O_{16}$	431(100), 269(42)		
30	eriodictin	9.85		433.1140	$C_{21}H_{22}O_{10}$	167(100), 197(75), 287(60)		
31	scutellarein-6,7-diglucuronide	9.9		637.1057	$C_{27}H_{26}$O18	285(100), 461(18)		

32	5,7,2',5'-tetrahydroxy-8,6'-dimethoxyflavone-glucose	10.0	215, 264, 330	507.1158	$C_{23}H_{24}O_{13}$	345(100), 330(80)		
33	8-C-glucose-6-C-arabinose-chrysin	10.1	215, 275, 315	547.1470	$C_{26}H_{28}O_{13}$	167(100), 197(54), 287(15)	549.1597	351(100), 271(35), 179, 167
34	methyl hesperidin	10.2		623.1993	$C_{29}H_{36}O_{15}$	461(100), 315(13)		
35	kaempferide 3-glucuronide	11	208, 276, 329	475.0886	$C_{22}H_{20}O_{12}$	299(100), 284(50)		
36	salviaflaside	11.2		521.1308	$C_{24}H_{26}O_{13}$	359(100)		
37	unknown	11.4		637.2119		461(50), 175(100), 315(21), 160(28)		
38	unknown	11.45		671.2935	$C_{32}H_{48}O_{15}$	509(100), 347(25), 389(18)		
39	hesperetin-7-O-β-D-glucuronide	11.5		477.1040	$C_{22}H_{22}O_{12}$	301(100), 286(28), 181(14)		
40	5,7,2',5'-tetrahydroxy-8,6'-dimethoxyflavone	11.7	215, 265, 338	345.0607	$C_{17}H_{14}O_{8}$	315(100), 330(15), 316(14), 164(6.5)	347.0759	289(100), 314(67), 317(53), 169(39), 150(27)
41	{2-[5,7-dihydroxy-2-(4-hydroxyphenyl)-4-oxo-chromen-8-yl]-4,5-dihydroxy-6-(hydroxymethyl)oxan-3-yl}oxidanesulfonic acid	11.9		511.0557	$C_{21}H_{20}O_{13}S$	269(100), 431(28)		
42	7-hydroxy-3-(2,4,5-trihydroxyphenyl)-3,4-dihydro-2H-1-benzopyran-4-one	12.3		287.0567	$C_{15}H_{12}O_{6}$	125(100), 161(50), 201(16)		
43	baicalin[a]	12.7	225, 280, 315	445.0771	$C_{21}H_{18}O_{11}$	269(100), 251(8)	447.0922	271(100), 253(2)
44	dihydrobaicalin	12.8	215, 290	447.0929	$C_{21}H_{20}O_{11}$	271(100), 243(58), 253(13), 244(97)	449.1076	273(100), 169(12), 123(8), 131, 103
45	kaempferide diglucuronide	12.9	217, 285, 328	651.2297	$C_{30}H_{36}O_{16}$	175(100)475(11)193(21)160(18)299		
46	5,7,-dihydroxy-6-methoxy flavone-7-O-β-D-glucoside	13.4	220, 280	445.1137	$C_{22}H_{22}O_{10}$	267(100)283(5)	447.0922	285(100), 270(45), 252(2)
47	norwogonin-7-O-β-D-glucuronide	13.7	215, 280, 360	445.0771	$C_{21}H_{18}O_{11}$	269(100)	447.0926	271(100), 285(3.8)
48	5,6,7-trihydroxy-8-methoxy flavone-7-O-β-D-glucuronide	13.8	215, 284	475.0879	$C_{22}H_{20}O_{12}$	284(100)299(16.7)285(13)300,283	477.1029	286(100), 301(97), 269
49	oroxylin A 7-O-β-D-glucuronide	13.9	210, 270, 310	459.0928	$C_{22}H_{22}O_{11}$	268(100)283(16)269(13)	461.1084	270(100), 285(73)
50	chrysin-glucuronide	14.0	270, 310	429.0821	$C_{21}H_{18}O_{10}$	253(100)	431.0975	255(100)
51	5,7,8-trihydroxy-6-methoxy flavone-7-O-β-D-glucuronide	14.2	210, 290	475.0879	$C_{22}H_{22}O_{12}$	284(100)299(33)285(13)300(4.5)	477.1030	286(100), 301(88), 184(4.3)
52	wogonoside[a] (wogonin-7-O-β-D-glucuronide)	14.4	220, 275, 345	459.0929	$C_{22}H_{22}O_{11}$	268(100)283(15)269(13)284(2.2)	461.1085	270(100), 285(85)
53	5,7-dihydroxy-6,8-dimethoxyflavone-7-O-β-D-glucuronide	14.6	220, 280	489.1035	$C_{23}H_{22}O_{12}$	298(100)283(45)299(14)313(13)	491.1191	315(100), 285(85),

								300(36), 282(28)
54	norwogonin	14.8	215, 270	285.0413	$C_{15}H_{10}O_6$	268(100)151(15)		
55	5,7,4′-trihydroxy-6-methoxy flavone	15,0	217, 286	301.0727	$C_{16}H_{14}O_6$	286(100),165(74), 181(24),137(17),258(8),230(4)		
56	5,2′,5′-trihydroxy-6,7,8-tri-methoxyflavone	15.3	217, 285	359.0787	$C_{18}H_{16}O_8$	329(100), 344(47), 314(21), 194(8)		
57	apigenin	15.6	220, 280	269.0453	$C_{15}H_{10}O_5$	197(100), 171(40), 213(31)	271.0604	169(100), 139(12), 123(9), 141(8)
58	5,6-dihydroxy-6-methoxyfla-vone	15.9	220, 280, 325	299.0561	$C_{16}H_{12}O_6$	284(100), 181(14), 153(14), 285(13), 200(11)	301.0709	286(100), 184(80), 156(15), 137(5), 169(5)
59	baicalein[a]	16.2	215, 275, 320	269.0457	$C_{15}H_{10}O_5$	223(24), 241(20), 195(17), 169(15), 197(12), 251(11), 271(8)	271.0603	123(79), 253(21), 169(20), 103(8)
60	skullcapflavone derivative	16.6	219, 275, 325	423.0397		343(100), 328(62), 313(40), 298(20), 269(12)		
61	wogonin[a]	19.0	210, 275	283.0616	$C_{16}H_{12}O_5$	268(100), 163(23), 184, 239	285.0753	270(100), 179(8), 252(7.2), 242, 168
62	chrysin	19.3	220, 270, 310	253.0510	$C_{15}H_{10}O_4$	209	255.0648	153(63), 129, 147, 152, 103
63	5,2'-dihydroxy-6,7,8,6'-tetra-methoxyflavone (skullcapfla-vone II)	19.4	220, 270, 320	373.0934	$C_{19}H_{18}O_8$	343(100), 328(64), 300(24), 194(10), 358	375.1075	345(100), 197(54), 327(32), 169(12), 227(9)
64	oroxylin A[a]	19.6	215, 270, 320	283.0613	$C_{16}H_{12}O_5$	268(100), 165(8.8), 184(7), 239	285.0754	270(100), 168(46), 140(9), 242(7.5), 224(3)
65	4',5-dihydroxy-6,7,8-tri-methoxyflavone	20.3	220	343.0821	$C_{18}H_{16}O_7$	313(100), 298(67), 270(18.6), 299(9), 328, 194	345.0966	315(100), 197(42), 297(32), 169(11), 287(9)

Table S7. Two-way ANOVA with Tukey's multiple comparison test results for *S. baicalensis* root profile analysis.

Subgroups compared	Mean Diff.	95.00% CI of diff.	Significant?	Summary	Adjusted p value
**Wogonoside**					
Control vs. Xyl_1x	210.7	97.79 to 323.6	Yes	****	<0.0001
Control vs. Xyl_2x	785.4	672.5 to 898.3	Yes	****	<0.0001
Control vs. Xyl_3x	1489	1376 to 1602	Yes	****	<0.0001
Control vs. Glc_1x	-1040	-1153 to -926.8	Yes	****	<0.0001
Control vs. Glc_2x	1488	1375 to 1601	Yes	****	<0.0001
Control vs. Glc_3x	3648	3535 to 3761	Yes	****	<0.0001
Control vs. EtGly_1x	2863	2750 to 2976	Yes	****	<0.0001
Control vs. EtGly_2x	4094	3981 to 4206	Yes	****	<0.0001
Control vs. EtGly_3x	4476	4363 to 4589	Yes	****	<0.0001
Control vs. H2O_1x	699.9	587.0 to 812.8	Yes	****	<0.0001

Control vs. H2O_2x	356	243.1 to 468.9	Yes	****	<0.0001
Control vs. H2O_3x	1036	922.7 to 1149	Yes	****	<0.0001
**Wogonin**					
Control vs. Xyl_1x	-4.046	-116.9 to 108.9	No	ns	>0.9999
Control vs. Xyl_2x	-11.14	-124.0 to 101.8	No	ns	>0.9999
Control vs. Xyl_3x	-7.058	-120.0 to 105.8	No	ns	>0.9999
Control vs. Glc_1x	2	-110.9 to 114.9	No	ns	>0.9999
Control vs. Glc_2x	7.184	-105.7 to 120.1	No	ns	>0.9999
Control vs. Glc_3x	-22.07	-135.0 to 90.83	No	ns	>0.9999
Control vs. EtGly_1x	7.798	-105.1 to 120.7	No	ns	>0.9999
Control vs. EtGly_2x	-31.77	-144.7 to 81.13	No	ns	0.9991
Control vs. EtGly_3x	11.54	-101.4 to 124.4	No	ns	>0.9999
Control vs. H2O_1x	1.768	-111.1 to 114.7	No	ns	>0.9999
Control vs. H2O_2x	-17.62	-130.5 to 95.28	No	ns	>0.9999
Control vs. H2O_3x	-3.412	-116.3 to 109.5	No	ns	>0.9999
**Baicalin**					
Control vs. Xyl_1x	-40.47	-153.4 to 72.43	No	ns	0.9913
Control vs. Xyl_2x	73.63	-39.27 to 186.5	No	ns	0.5853
Control vs. Xyl_3x	212.7	99.81 to 325.6	Yes	****	<0.0001
Control vs. Glc_1x	-40.57	-153.5 to 72.33	No	ns	0.9911
Control vs. Glc_2x	119.2	6.332 to 232.1	Yes	*	0.0286
Control vs. Glc_3x	478.7	365.8 to 591.6	Yes	****	<0.0001
Control vs. EtGly_1x	357.6	244.7 to 470.5	Yes	****	<0.0001
Control vs. EtGly_2x	539.7	426.8 to 652.6	Yes	****	<0.0001
Control vs. EtGly_3x	594.3	481.4 to 707.2	Yes	****	<0.0001
Control vs. H2O_1x	42.57	-70.33 to 155.5	No	ns	0.9866
Control vs. H2O_2x	-14.71	-127.6 to 98.19	No	ns	>0.9999
Control vs. H2O_3x	48.5	-64.39 to 161.4	No	ns	0.9621
**Baicalein**					
Control vs. Xyl_1x	-14.84	-127.7 to 98.06	No	ns	>0.9999
Control vs. Xyl_2x	-4.317	-117.2 to 108.6	No	ns	>0.9999
Control vs. Xyl_3x	0.5231	-112.4 to 113.4	No	ns	>0.9999
Control vs. Glc_1x	-7.896	-120.8 to 105.0	No	ns	>0.9999
Control vs. Glc_2x	11.92	-101.0 to 124.8	No	ns	>0.9999
Control vs. Glc_3x	20.32	-92.58 to 133.2	No	ns	>0.9999
Control vs. EtGly_1x	19.13	-93.77 to 132.0	No	ns	>0.9999
Control vs. EtGly_2x	19.65	-93.25 to 132.5	No	ns	>0.9999
Control vs. EtGly_3x	20.84	-92.06 to 133.7	No	ns	>0.9999
Control vs. H2O_1x	8.875	-104.0 to 121.8	No	ns	>0.9999
Control vs. H2O_2x	-23.22	-136.1 to 89.68	No	ns	>0.9999
Control vs. H2O_3x	5.407	-107.5 to 118.3	No	ns	>0.9999

The unsupervised method PCA (Figure S1, Figure S2) was applied to discover appropriate group patterns in the data and to examine whether the metabolites are also detected as being significant. This method is called "unsupervised" because it is performed without data imputation with class membership, meaning that it shows the main structure in

the data without considering a special direction or type of information. PCA is a powerful method to perform the dimension reduction of a data set containing thousands of metabolites, summary of them for specified component that best explain the total variation in the original data set. OPLS-DA (Figure S3, Figure S4) is a one of the "supervised" method that discriminates between groups of samples that are defined by the analyst, and show the signals giving maximum separation of this groups. In addition on orthogonal filter is involved into the OPLS algorythim, which allows to removal of spectral components unrelated to the sample classes chosen , i.e., filtering the markers from the biological varieties.

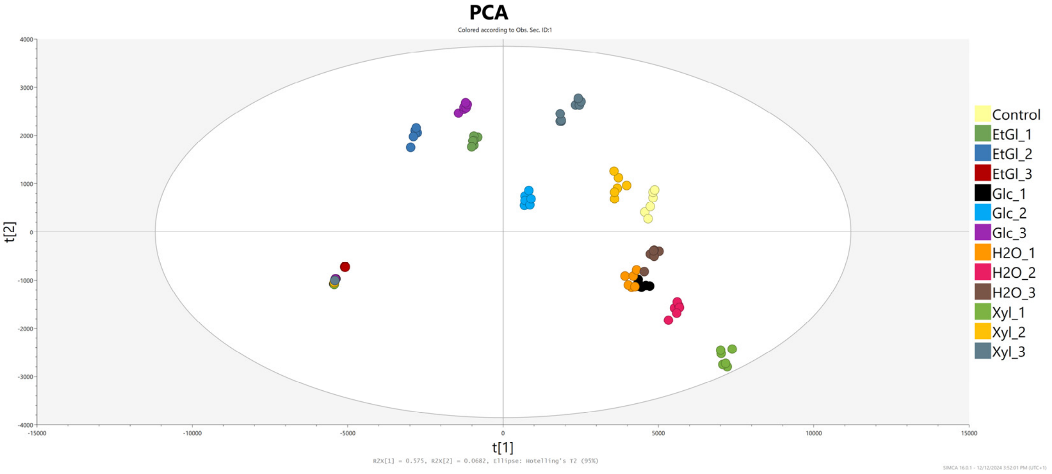

Figure S1. PCA analysis of Sb root extracts prepared three weeks after PEF treatment (R2XCum =0.882, Q2Cum=0.798). Scores plot for each of the treated subgroups and Control.

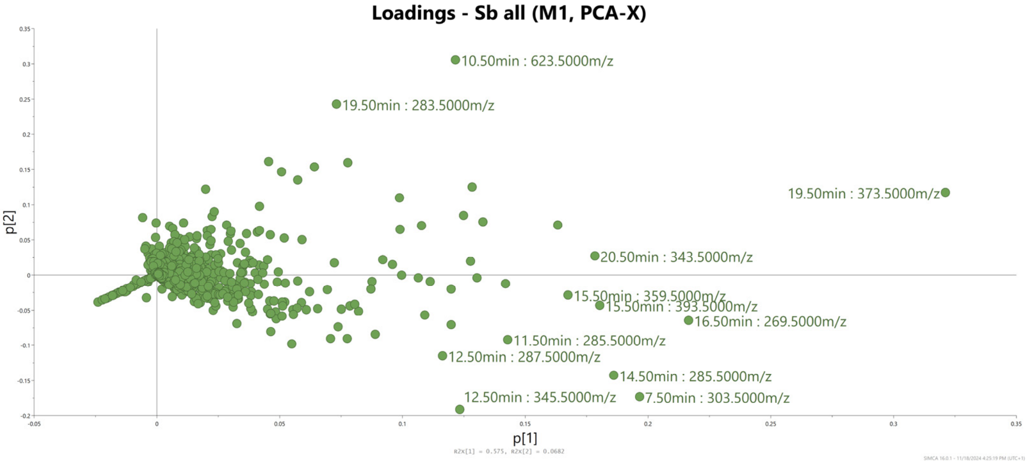

Figure S2. Loadings plot of the PCA model. Compounds of note which influence the model the most: 7.50 min, 303.5 m/z 3,5,7,2′,6′-pentahydroxyflavanone; 10.50 min, 623.5 m/z – methylhesperidin; 11.50 min, 285.5 m/z – scutellarein; 12.50 min, 345.5 m/z - 5,7,2′,5′-Tetrahydroxy-8,6′-dimethoxyflavone; 12.50 min, 287.5 m/z - 7-hydroxy-3-(2,4,5-trihydroxy-phe-nyl)-3,4-dihydro-2H-1-benzopyran-4-one; 14.50 min, 285.5 m/z – norwogonin; 15.50 min, 359.5 m/z – 5,2′,5′-trihydroxy-6,7,8-trimethoxyflavone; 15.50 min, 393.5 m/z - ?; 16.50 min, 269.5 m/z – baicalin; 19.50 min, 283.5 m/z – wogonin; 19.50 min, 373.5 m/z - skullcapflavone II; 20.50 min, 343.5 m/z - 4′,5-Dihydroxy-6,7,8-trimethoxyflavone.

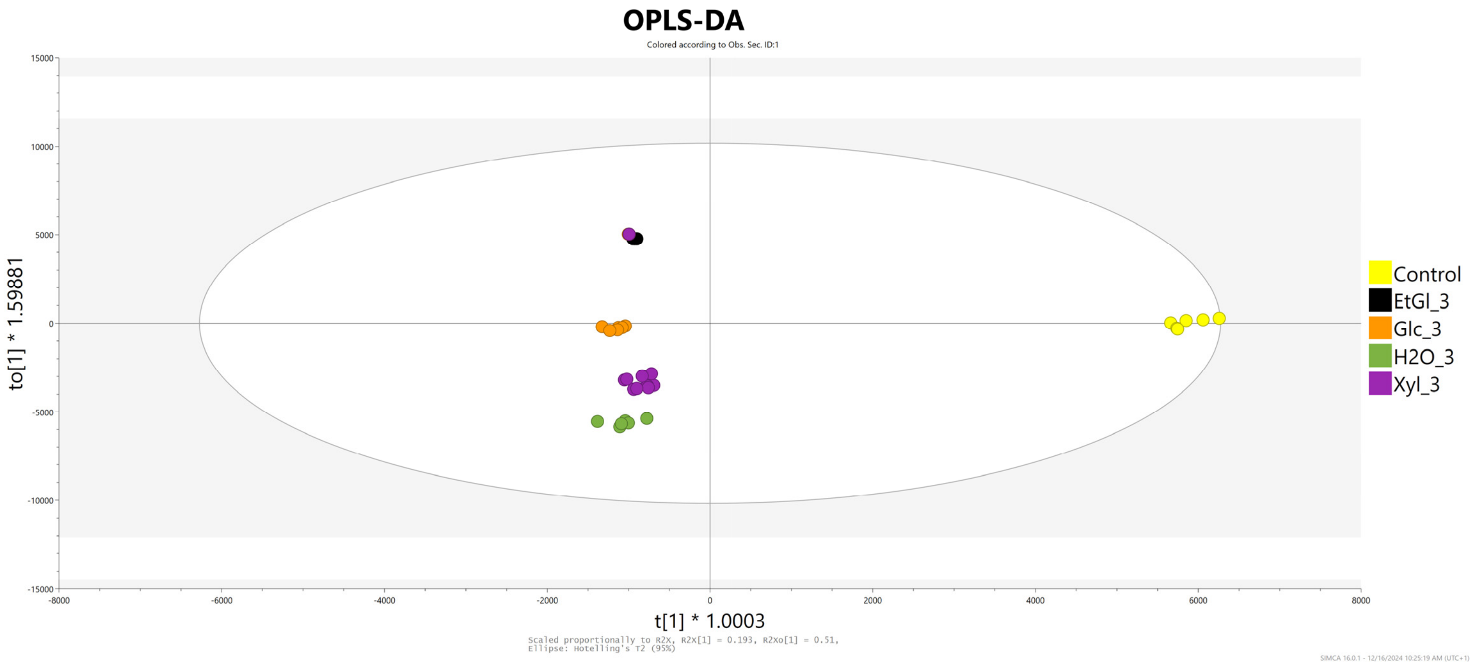

Figure S3. OPLS-DA analysis of Sb root extracts prepared three weeks after PEF treatment (R2XCum =0.716, R2Y (cum) 0.994, Q2Cum=0.954). Scores plot for subgroups that were treated exactly three times with PEF.

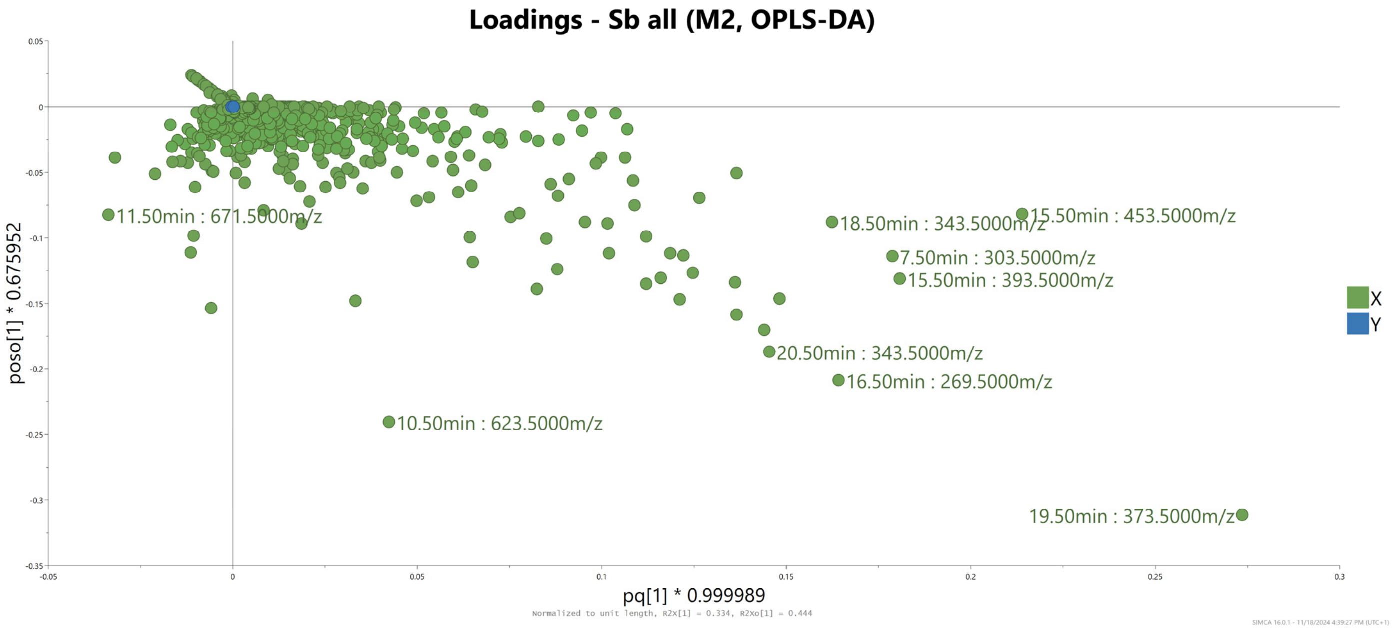

Figure S4. Loadings plot of the OPLS-DA model. Compounds of note which influence the model the most: 7.50 min, 303.5 m/z 3,5,7,2′,6′-pentahydroxyflavanone; 10.50 min, 623.5 m/z – methylhesperidin; 11.50 min, 671.5 m/z - ?; 15.50 min, 393.5 m/z - ?; 15.50 min, 453.5 m/z - ?; 16.50 min, 269.5 m/z – baicalin; 18.50 min, 343.5 m/z - ?; 19.50 min, 373.5 m/z - skullcapflavone II; 20.50 min, 343.5 m/z - 4′,5-Dihydroxy-6,7,8-trimethoxyflavone.

Disclaimer/Publisher's Note: The statements, opinions and data contained in all publications are solely those of the individual author(s) and contributor(s) and not of MDPI and/or the editor(s). MDPI and/or the editor(s) disclaim responsibility for any injury to people or property resulting from any ideas, methods, instructions or products referred to in the content.
